# The Physiological Molecular Shape of Spectrin: A Compact Supercoil Resembling a Chinese Finger Trap

**DOI:** 10.1371/journal.pcbi.1004302

**Published:** 2015-06-11

**Authors:** Jeffrey W. Brown, Esther Bullitt, Sira Sriswasdi, Sandra Harper, David W. Speicher, C. James McKnight

**Affiliations:** 1 Department of Physiology and Biophysics, Boston University School of Medicine, Boston, Massachusetts, United States of America; 2 Internal Medicine Residency Program, University of Pittsburgh Medical Center, UPMC Montefiore Hospital, Pittsburgh, Pennsylvania, United States of America; 3 Center for Systems and Computational Biology, and Molecular and Cellular Oncogenesis Program, the Wistar Institute, Philadelphia, Pennsylvania, United States of America; 4 Genomics and Computational Biology Graduate Group, University of Pennsylvania, Philadelphia, Pennsylvania, United States of America; Centre National de la Recherche Scientifique, FRANCE

## Abstract

The primary, secondary, and tertiary structures of spectrin are reasonably well defined, but the structural basis for the known dramatic molecular shape change, whereby the molecular length can increase three-fold, is not understood. In this study, we combine previously reported biochemical and high-resolution crystallographic data with structural mass spectroscopy and electron microscopic data to derive a detailed, experimentally-supported quaternary structure of the spectrin heterotetramer. In addition to explaining spectrin’s physiological resting length of ~55-65 nm, our model provides a mechanism by which spectrin is able to undergo a seamless three-fold extension while remaining a linear filament, an experimentally observed property. According to the proposed model, spectrin’s quaternary structure and mechanism of extension is similar to a Chinese Finger Trap: at shorter molecular lengths spectrin is a hollow cylinder that extends by increasing the pitch of each spectrin repeat, which decreases the internal diameter. We validated our model with electron microscopy, which demonstrated that, as predicted, spectrin is hollow at its biological resting length of ~55-65 nm. The model is further supported by zero-length chemical crosslink data indicative of an approximately 90 degree bend between adjacent spectrin repeats. The domain-domain interactions in our model are entirely consistent with those present in the prototypical linear antiparallel heterotetramer as well as recently reported inter-strand chemical crosslinks. The model is consistent with all known physical properties of spectrin, and upon full extension our Chinese Finger Trap Model reduces to the ~180-200 nm molecular model currently in common use.

## Author Summary

Spectrins are cytoskeletal and scaffolding proteins ubiquitously expressed in essentially all cell-types. Despite unequivocal evidence for a short physiological length of ~55–65 nm at rest, spectrin is typically represented as an extended ~200 nm molecule that is implied based on crystallographic structures of a number of tandem repeats. Here, we incorporate previously reported biochemical and crystallographic data with structural mass spectroscopy and electron microscopic data to derive a detailed, experimentally-supported quaternary structure of the physiological compact form of spectrin. In addition to explaining spectrin’s physiological resting length (~55–65 nm), our model provides a mechanism by which spectrin can undergo a seamless three-fold extension, which is an experimentally observed property that is responsible for restoration of cell shape after mechanical deformation.

## Introduction

**Fig 1:**
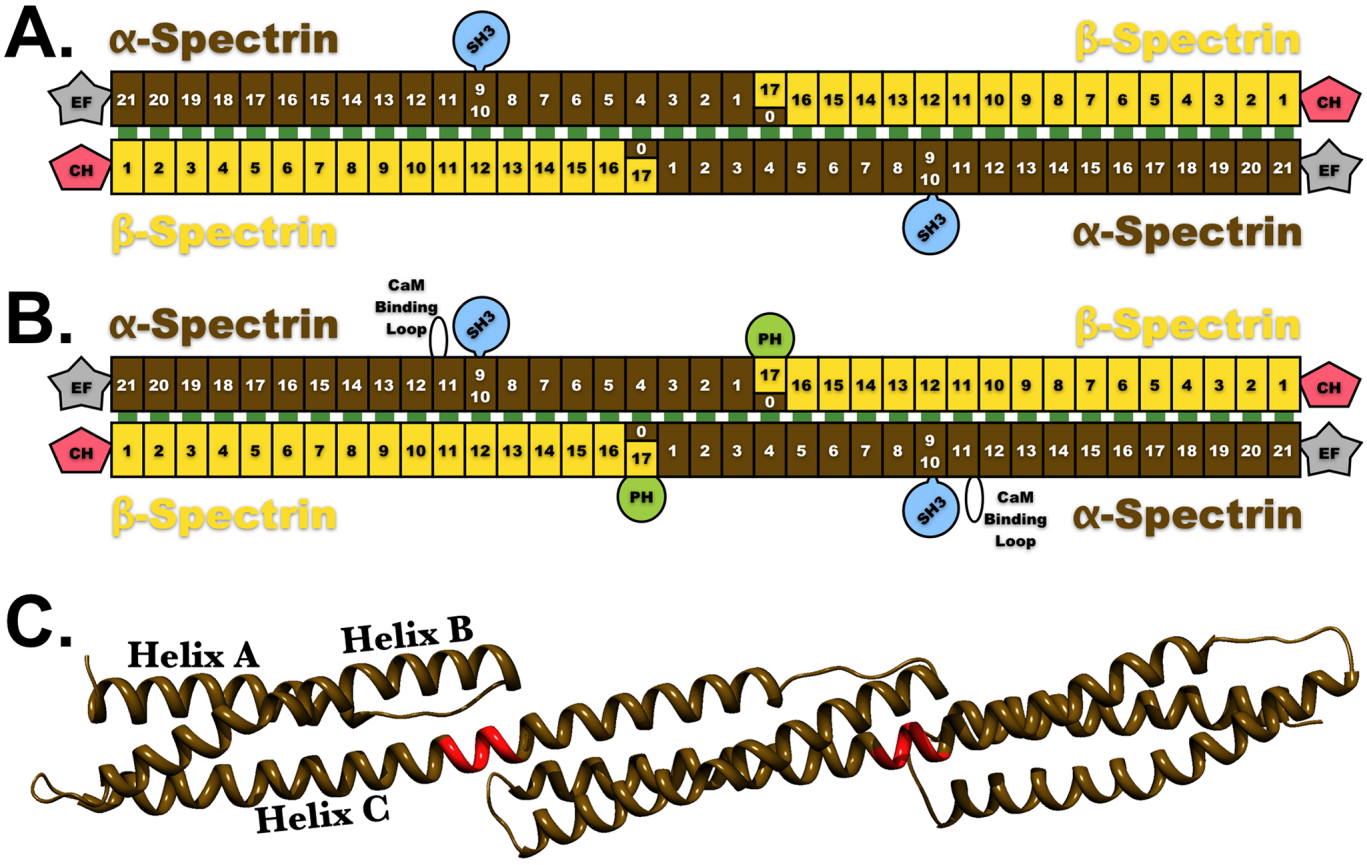
Spectrin domain structure. Cartoon representation depicting the domain organization of (**A**) erythroid and (**B**) nonerythroid spectrin tetramers (α-spectrin—brown spectrin repeats; β-spectrin—yellow spectrin repeats). The pink pentagons labeled CH are the actin binding domains other known spectrin domains are labeled EF, SH3 and PH. **C**. Ribbon representation of three consecutive spectrin repeats (brown); the 5-residue linker regions between each repeat are colored red.

Spectrins are 200+ kDa filamentous proteins present in most cell types; although they are best known for their role in the hexagonal arrangement of junctional complexes in erythrocytes and of microvilli in enterocytes. Humans and other mammals express a number of spectrin isoforms produced from multiple genes as well as by alternative gene splicing. The primary sequences of spectrins are mostly comprised of many tandem homologous domains that are ~106 residues in length and are commonly termed “spectrin repeats”. A number of high-resolution structures have been reported for both individual as well as several tandem spectrin repeats. All crystal structures exhibit a similar left-handed, anti-parallel, three-helix coiled-coil topology with short helical connectors between adjacent repeats [1–11]. Spectrin polypeptides are grouped into two categories: (1) α-spectrins, which contain 20 full spectrin repeats plus several other structural motifs and (2) β-spectrins, which contain 16 or more full spectrin repeats, an N-terminal actin-binding domain and a C-terminal nonhomologous domain of varying size. The biologically relevant form of spectrin is typically the heterotetramer formed through the antiparallel lateral association of an α-chain with a β-chain to form the heterodimer, which further dimerizes in a self-limiting, head-to-head fashion with another heterodimer to form the heterotetramer (Fig 1). One common function of the spectrin family of proteins is to act as a versatile actin crosslinker in diverse cell types.

The spectrin tetramer is often still cited as being ~200 nm in length and most models published over the past decade continue to represent the molecule in this manner. However, several lines of evidence, including direct *in situ* visualization, demonstrate its biological functional length is much shorter at rest (~55–65 nm), which we will subsequently refer to as the “compact form.” The ~200 nm “extended form” is now generally thought to result from either tensile stretching (*in vivo* or *in vitro*) or as a consequence of certain *in vitro* isolation conditions. Conditions reported to favor the extended form include the use of low ionic strength solutions, low protein concentration (reducing macromolecular crowding effects), to a small extent reduced temperature, and possibly removal of the associated membrane [12]. The simplest evidence for the shorter physiological length is predicated on the numerical density of spectrin tetramers or junctional complex components per surface area of erythrocyte membrane (Table 1). Using a consensus value of 10^5^ spectrin tetramers per erythrocyte [13] and employing a homogeneous hexagonal distribution across the erythrocyte membrane with a surface area of 135 μm^2^, one calculates a length of ~65 nm for each spectrin tetramer [13]. Similar values are obtained using other estimates of spectrin, ankyrin, actin, or other components of the junctional complex (S1 Table). Shorter lengths indicative of the compact form have been directly observed with both electron [14–17] and atomic force [18,19] microscopic examination of erythrocytes. Interestingly, the brain isoform of spectrin, which is known to be stiffer than other spectrin isoforms, appears to exist as the extended form *in vivo* [20] at least in some cell types.

**Table 1:** Length of the spectrin tetramer computed from the protein composition of the erythrocyte assuming a hexagonal cytoskeletal geometry.

Protein	Copies per Erythrocyte	Number per Junctional Complex	Computed Length (nm)	Reference
Actin	340,000	14	80.1	Fairbanks *et al*., 1971 [61]
Actin	360,000	14	77.9	Steck, 1974 [13]
Actin	500,000	14	66.1	Pinder & Gratzer, 1983 [62]
Adducin	30,000 Dimers	1	72.1	Gardner & Bennett, 1986 [63]
Ankyrin	100,000	-	68.4	Bennett, 1990 [64]
Ankyrin	124,500	-	61.3	Savvides *et al*., 1993 [65]
Dematin	129,000	6	85	Husain-Chishti *et al*., 1988 [66]
Spectrin	85,000–115,000	-	68.4	Fairbanks *et al*., 1971 [61]
Spectrin	108,000	-	65.8	Steck, 1974 [13]
Spectrin	100,000	-	68.4	Pinder & Gratzer, 1983 [62]
Spectrin	133,500	-	59.2	Shelton *et al*., 1984 [67]
Spectrin	121,000	-	62.2	Savvides *et al*., 1993 [65]
Tropomyosin	70,000–80,000	2	64.5	Fowler & Bennett, 1984 [68]
Tropomodulin	30,000	1	72.1	Fowler, 1987 [69]

Please refer to S1 Table for details concerning this computation.

The extraordinary level of reversible membrane deformability exhibited by erythrocytes as they traverse small capillaries has been attributed primarily to spectrin’s high degree of extensibility, for which four mechanisms have been proposed. The first involves a transformation from a more or less chaotic arrangement of spectrin repeats in the compact molecular form into a linear antiparallel arrangement upon extension [21,22]. Although this would act to shorten spectrin’s effective length, it is in disagreement with electron microscopic examinations of erythrocytes [17,23] and enterocytes [24], which demonstrate that spectrin tetramers are linear and that the diameter of each spectrin molecule does not fluctuate along its length. Second, it has been proposed that, in an analogous manner to titin [25], reversible unfolding of individual spectrin repeats could explain spectrin’s elasticity [26,27]. Although this mechanism might be relevant under conditions of extreme tensile deformation, unfolding of spectrin repeats seems unlikely to occur prior to first stretching the tetramer to the ~200 nm extended form. A third mechanism, termed the Extensible Linked Mosaic Model [3], is based on x-ray crystal structures which suggested that the spectrin repeat is able to slide up and down the primary sequence like an overhand knot along a piece of string. Although, this is an intriguing mechanism, it has only been observed subsequent to mutagenesis of the highly conserved leucine residues that flank the linker region (S1 Fig) and does not accurately reproduce either the diameter or the periodicity experimentally measured by others [15,23]. The fourth mechanism, predicated on the direct electron microscopic examination of partially stretched erythrocytic membranes, suggests that spectrin is organized as a two-start right-handed helix and functions like a tension spring [23]. We favor this later mechanism as it most closely fits known physical and imaging data. Therefore, we combine the previously reported biochemical, microscopic, and crystallographic data with our structural mass spectroscopy and electron microscopic data to derive a new model of spectrin’s molecular form that explains its morphology and provides a mechanism of extension and contraction.

## Results

### The Crystallographic Structure of Spectrin

Spectroscopic and crystallographic studies have established that both α- and β-spectrins fold into a tandem series of topologically identical domains, individually referred to as spectrin repeats (Fig 1). Further, the head-to-head tetramerization event results in the formation of an additional spectrin repeat, that will be referred to, here, as the “tetramerization repeat” and which is created through the association of a partial repeat near the C-terminus of β-spectrin (helices A and B) with a partial repeat near the N-terminus of α-spectrin (helix C) [10,11]. Through this tetramerization repeat, an alpha spectrin associates with a beta spectrin to form an uninterrupted chain of 37 repeats. In an analogous manner to that implemented in Kusunoki *et al*., 2004 [5], we first created a model of the spectrin heterotetramer from x-ray crystal structures (S2 Fig). This model is morphologically similar to the rotary shadowed electron micrographs of isolated spectrin tetramers [28]. While this crude model resembled rotary shadowed EM images, the absence of lateral association between α and β chains along most of their lengths is inconsistent with chemical crosslinking data from spectrin in solution and on intact red cell membranes [29–31]. Further, this concatenation of x-ray crystal structure assumes a gentle left-handed supercoil, while electron microscopy demonstrated that the native quaternary structure of spectrin is a right-handed supercoil [23]. Lastly, at ~180–200 nm long, this “Crystallographic Model” of the spectrin tetramer is inconsistent with spectrin’s biologically functional length (~55–65 nm). This led us to conclude that, in vivo, the spectrin tetramer must be conformationally different than models based on simple concatenation of crystallographic structures of dimeric spectrin repeats.

### The Linker Region

**Fig 2:**
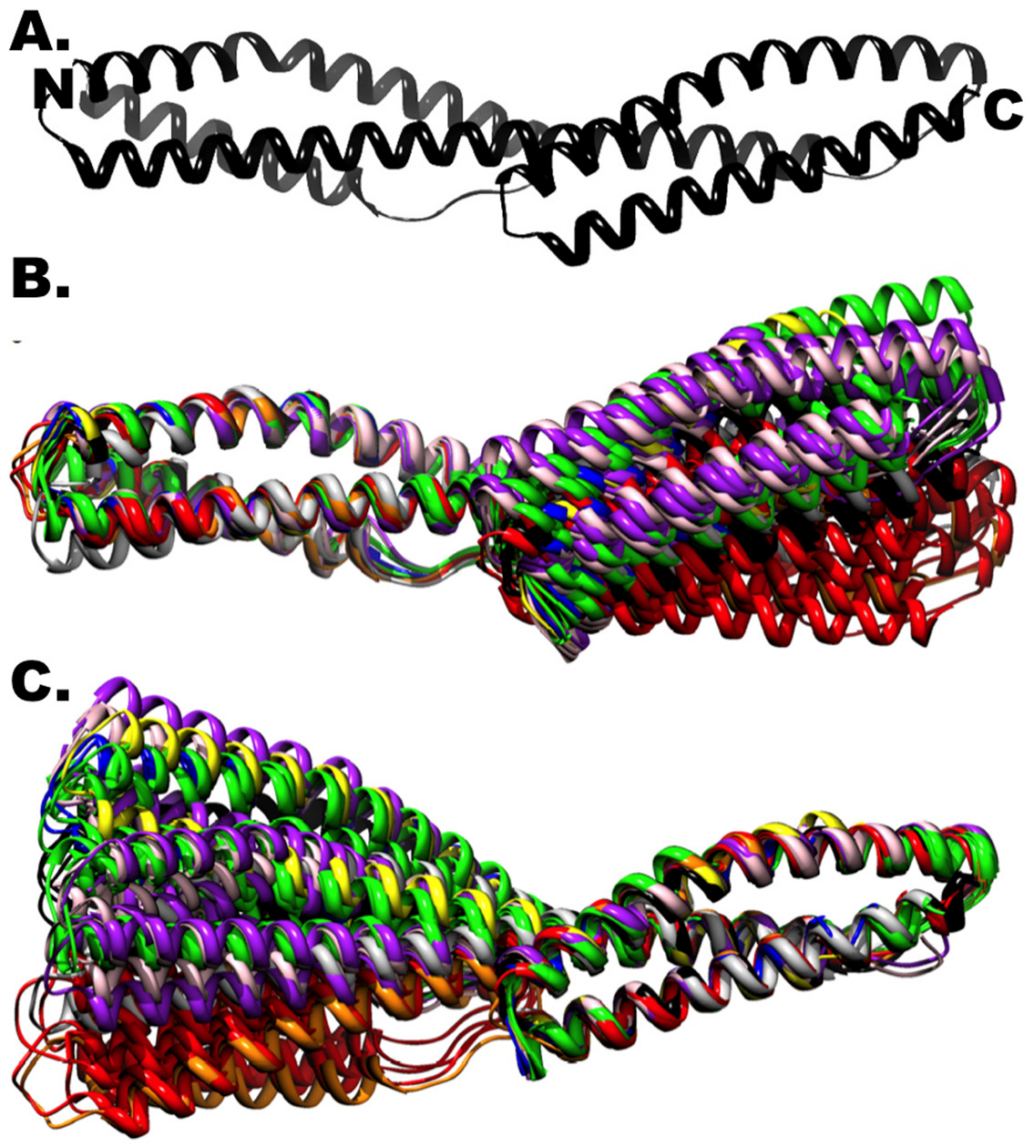
Structural alignment of spectrin di-repeats. (**A**) Ribbon representation of a spectrin direpeat. All currently available high resolution structures of tandem spectrin repeats (from spectrin, excluding homologs) are aligned to either the N-terminal or C-terminal repeat in (**B**) and (**C**), respectively. (PDB Accession IDs: 1CUN, black; 1S35, grey; 1U4Q, red; 1U5P, orange; 3EDU, yellow; 3EDV, green; 3F57, blue; 3KBT, purple; 3KBU, pink).

Although no high-resolution data currently exists that explains spectrin’s ~55–65 nm resting length, superimposition of all published x-ray crystal structures suggests a mechanism by which spectrin assumes this shorter length. In Fig 2, we display our results of a sequence independent, C_α_-based structural alignment of all published tandem spectrin direpeats to either its N-terminal or C-terminal repeat (Fig 2B and 2C, respectively). As each spectrin repeat aligns well at the expense of its adjacent domain and the linker region, we conclude that, unlike the fold of each spectrin repeat which is structurally invariant, the conformation of the 5-residue region linking each pair of repeats (appropriately termed the linker region) varies significantly between crystal structures and even between asymmetric molecules within a single crystallographic unit cell [6].

From our initial crystallographic model of the spectrin heterotetramer (S2 Fig), we demonstrated that a continuous alpha helix between Helix C of the i^th^ repeat and Helix A of the i^th^+1 repeat results in a spectrin tetramer with length similar to the maximum length observed of spectrin, which is three times longer than its biological resting length. There is no available evidence to suspect drastic conformational changes within the spectrin repeat as it transitions from the compact to the extended form. Therefore, we propose that the dynamic linker region between spectrin repeats remodels to produce a quaternary structure of spectrin that is consistent with the two-start helix reported by McGough and Josephs [23].

### The Chinese Finger Trap Model

**Fig 3:**
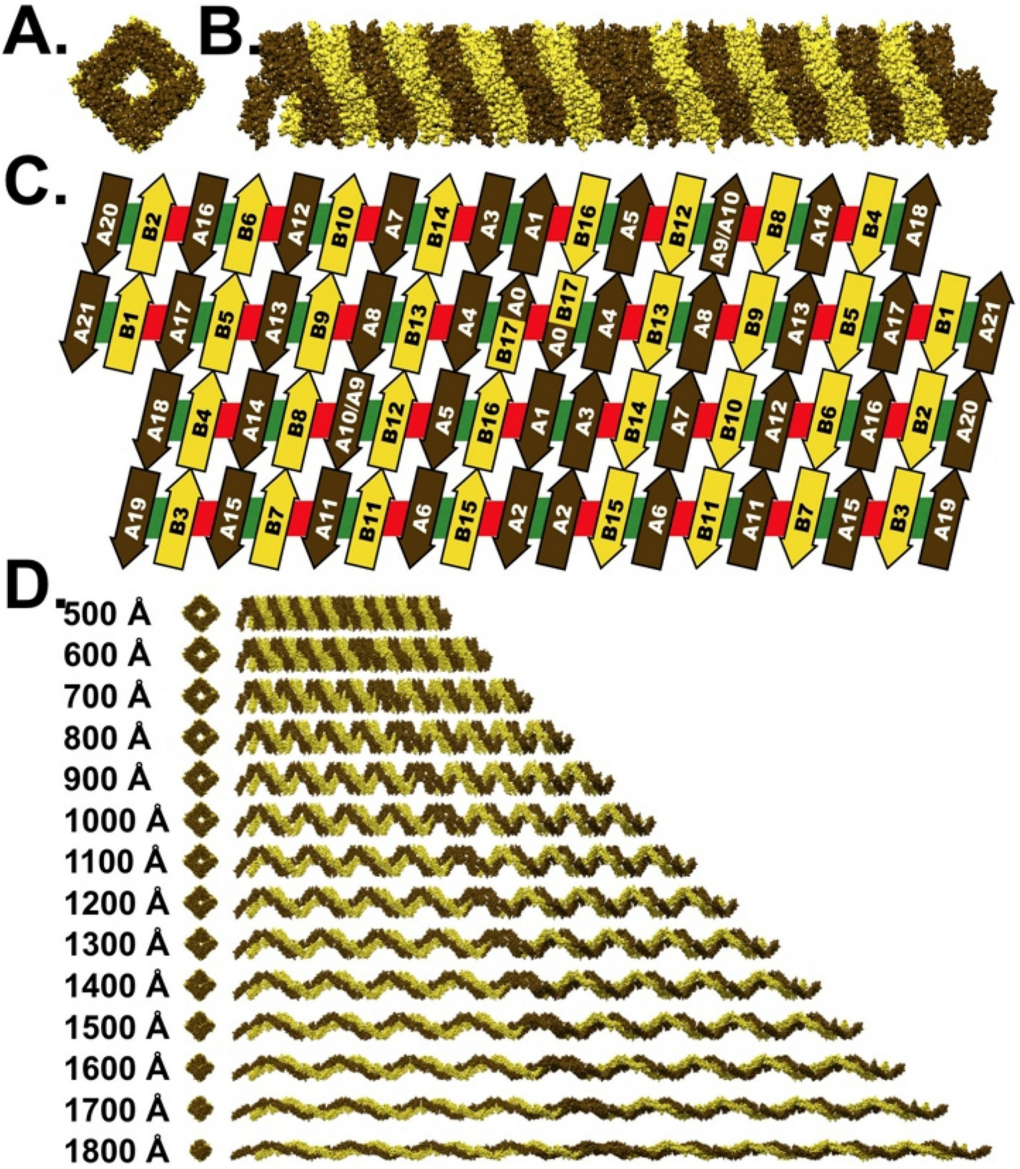
The “Chinese Finger Trap” model of spectrin. **A** End View, and **B**. Side Views of the spectrin repeats that comprise each tetramer are arranged in a similar fashion to the geometry reported by McGough & Josephs, 1990 (See Materials and Methods). **C**. Schematic representation of all interdomain interactions present within the Chinese Finger Trap model. The green bars indicate all interactions present within both the extended and Chinese Finger Trap model, while the red bars identify the interactions predicted to exist only in the Chinese Finger Trap Model. **D**. The proposed mechanism by which spectrin can undergo a three fold extension, while remaining a linear filamentous polymer.

Spectrin’s contour length (defined in S3 Fig) was experimentally determined to be 19.9 nm and, like a spring, was invariant to either contraction or extension [23]. Intriguingly, this value is precisely the length of four spectrin repeats (~5 nm per repeat including one linker region) and, therefore, we, as did McGough & Josephs (but for other reasons), propose that four spectrin repeats comprise each supercoil turn. This quaternary conformation is obtained by a 90° bend between tandem spectrin repeats (Fig 3A), a conformation that we experimentally observe in mini-spectrin tetramers (see below). In the resulting model of the spectrin tetramer (Fig 3), the two, antiparallel strands are phased so that the high affinity interaction between α_20,21_ and β_1–2_ is maintained and arranged in accord with inter-strand chemical cross-linking data [29–35]. A striking prediction from this level of modeling is that spectrin is a hollow cylinder at its biological resting length (Fig 3A).

Comparison of both the domain organization as well as domain-domain interactions present within the Extended Model (Fig 1A and 1B) to those in our Chinese Finger Trap Model (Fig 3C) demonstrates the complete conservation of all intra- and interdomain interactions. As a result, all known properties of spectrin domain interactions (green bars in Figs 1A and 3C) and alignments are consistent with our Chinese Finger Trap Model. Furthermore, our model predicts potential new interdomain interactions, which are schematically represented as red bars in Fig 3C. The Chinese Finger Trap Model is formed by supercoiling the Extended Model. When the Chinese Finger Trap Model is extended, it reduces to a supercoiled configuration of the Extended Model (Fig 3D). Thus, these two models are not mutually exclusive, but instead represent two extremes along a dynamic continuum of spectrin’s quaternary structure (Fig 3D).

### A 90° Bend in the Linker Region

**Fig 4:**
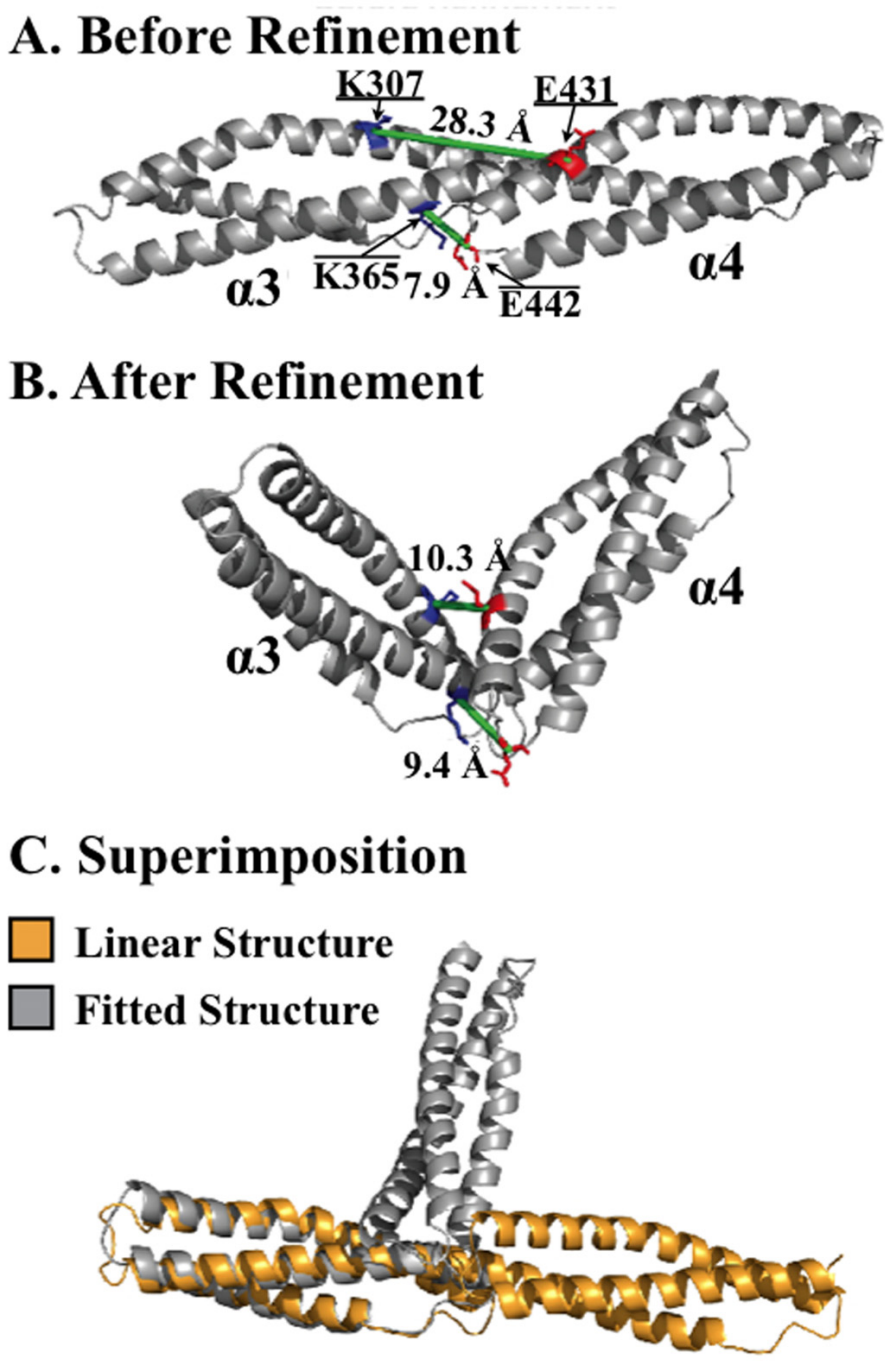
Refinement of a spectrin di-repeat using structural mass spectroscopy. **A**. Two observed high confidence zero-length crosslinks (green lines) were mapped onto the extended structure of the spectrin α3–4 region of mini-spectrin. Crosslinked residues are highlighted in blue (lysine) and red (acidic residue) and alpha carbon distances are shown. **B.** Refinement of the above structure by allowing connector residues to be non-helical and using distance constraints for the two inter-repeat crosslinks. **C.** Superimposition of the two structures.

The extremely large (~1 megadalton) size and dynamic nature of the native spectrin tetramer makes identification of individual residue-residue interactions extremely difficult. To circumvent these difficulties, a “mini-spectrin” construct was created that retains many of the properties of the spectrin tetramer in a construct that can be expressed, mutated, and studied structurally and functionally [30]. Unfortunately, as with other spectrin constructs larger than four repeats, mini-spectrin dimers and tetramers failed to produce X-ray diffraction quality crystals. Hence, structural mass spectrometry and molecular modeling were used to produce medium resolution structures of the extended form of mini-spectrin tetramers and dimers [29]. In that study, only the extended form of the protein was modeled because the crystal structure templates used for modeling contained helical linker domains. Interestingly, datasets from crosslinking mini-spectrin tetramers using a zero-length crosslinker contained two high confidence crosslinks between the α3 and α4 domains that were inconsistent with a linear, helical linker domain (K307-E431, K365-E442; Fig 4). The mass spectrum of the critical K365-E442 cross-link is displayed in S4 Fig. When the linker region between the α3 and α4 repeats was allowed to adopt a non-helical conformation and distance constraints for the two crosslinked sites spanning the connector were used to refine the model, a ~90° bend between the natively structured α3 and α4 tandem spectrin repeats was observed. This bend and the relative orientation of the tandem spectrin repeats are consistent with the Chinese Finger Trap Model described above.

### Spectrin Is Hollow at Its Physiological Compact Resting Length

**Fig 5:**
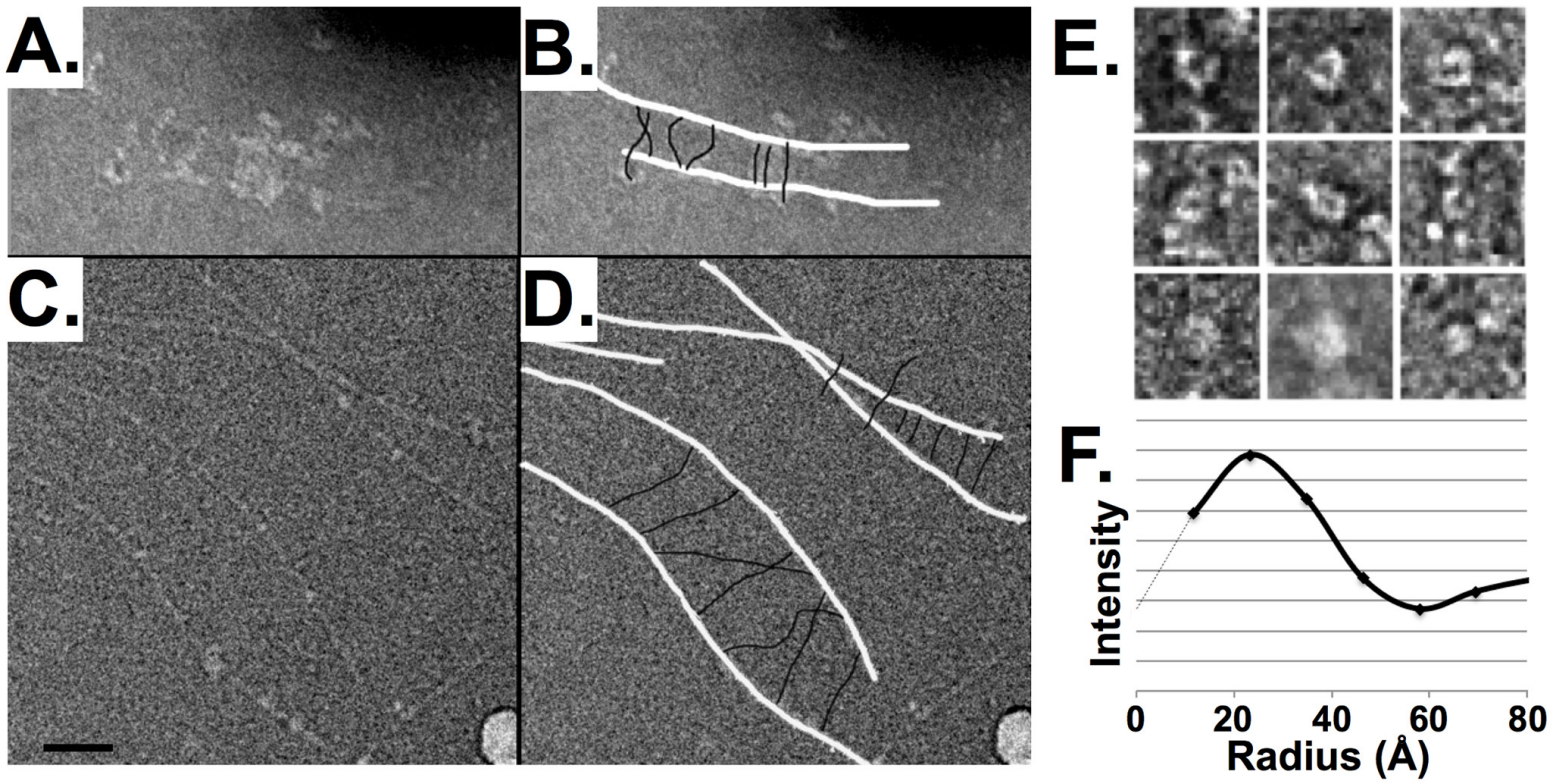
Electron microscopy of actin-spectrin ladders. **A**. Transmission electron micrograph (TEM) of negatively-stained actin and spectrin illustrating “ladders”, with actin as the ‘rails’ and spectrin as the ‘rungs’. **B**. Cartoon of actin and spectrin overlaid on the micrograph shown in panel A (actin: white, spectrin: black; not to scale). **C** and **D**. TEM and cartoon, respectively, of frozen-hydrated actin and spectrin, again illustrating the ladder-like appearance. Contrast of the cryoEM images is inverted so that protein is white, as in panels A, B and E. **E**. TEMs of negatively stained spectrin viewed end-on, in which some images show a hollow center. **F**. Radial density profile of a representative end-on view of spectrin; note that the center density (at r = 0) is low, and rises before falling off to the background value.

The molecular shape of spectrin was further analyzed using transmission electron microscopy (EM). Because we found spectrin to stain very poorly with several common contrast agents, we co-incubated spectrin with actin to better delineate the protein. The combination of these two proteins resulted in the formation of a ladder-like configuration that was visible in both negatively stained samples using EM (Fig 5A–5B), and unstained samples viewed using cryoEM (Fig 5C–5D). In these images, actin filaments are the rails and spectrin tetramers form the rungs of a ladder; this interpretation is supported by the presence of one spectrin binding site for each actin monomer, allowing numerous spectrin molecules to bind per filament, whereas spectrin has two F-actin binding sites so that one heterotetramer of spectrin can bind two actin filaments. Similar to EM examination of native preparations of spectrin [14–17], we found spectrin to be linear over a wide range of lengths (Fig 5).

In addition to actin/spectrin ladders, at much higher concentrations of spectrin (such that the grids were nearly saturated with spectrin) numerous spectrin tetramers were also visualized end-on, down the axis of the spectrin barrel (Fig 5E). This unique view was never observed at low concentrations and is believed to be the result of adjacent spectrin tetramers either propping up the barrel or bending the filament tip normal to the grid or potentially breakage of the spectrin filament into heterodimeric species, whose shorter length may make this species more amenable to lying normal to the grid (but still requiring support from adjacent spectrin tetramers). Plotting the electron density of these end-on views of spectrin as a function of the radial distance from the center clearly demonstrates the presence of a hollow core, as illustrated by the peak in intensity at ~2.2 nm produced by the negative stain in Fig 5F. This important property predicted by the Chinese Finger Trap model, has not been previously described. The ~2.2 nm radius of spectrin is half the length of a spectrin repeat sans the linker region (~4.5 nm based on available crystal structures), which is predicted by the Chinese Finger Trap model that has 4 spectrin repeats comprising each supercoil loop. Thus, our EM data further supports the Chinese Finger Trap model of spectrin.

### Interfaces between Sequential, Tandem Domains

**Fig 6:**
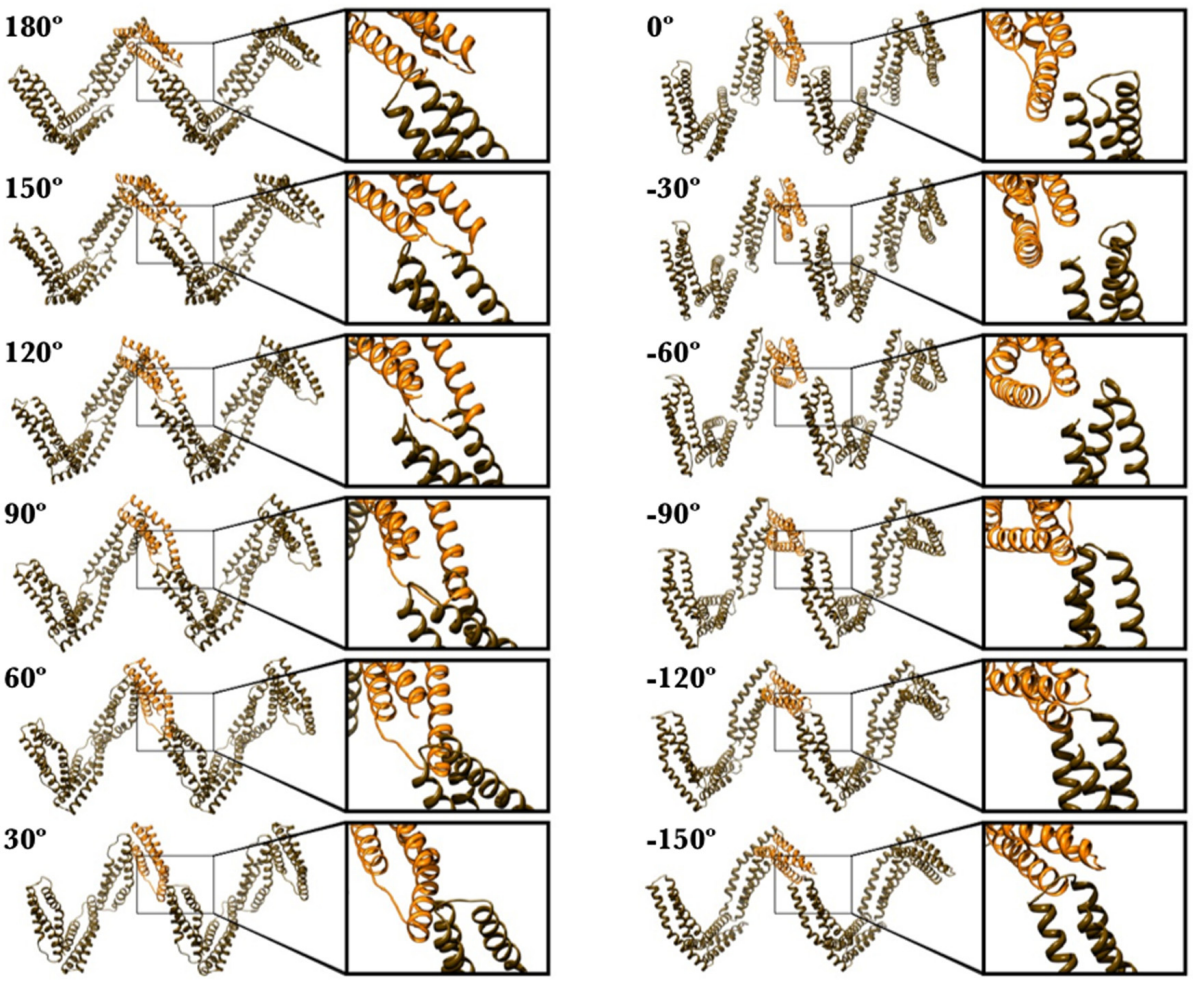
Using steric considerations to identify potential interdomain binding surfaces. Ribbon diagram of eight spectrin repeats (sans linker region) excised from our Chinese Finger Trap model at 65 nm. The difference between the 12 models depicted here is that each was calculated with a different “roll” parameter (refer to Materials and Methods, Fig 3). If each spectrin repeat is likened to a hotdog, the roll parameter would be equivalent to rotating the hotdog about a line through its long axis. Although the gross morphology of the hotdog would not change, if one focused on a single surface, that surface would rotate as the roll was incremented. Therefore, this roll parameter establishes which surfaces are involved in lateral interstrand interactions. The enlargements show that steric clashes arise between adjacent domains in all cases except those between the roll value of 0° and -60°.

Closer examination of the individual α-helices within the Chinese Finger Trap Model provides insight into the surfaces that interact between adjacent domains. In Fig 6, we display the effect of incrementing the “roll” (see Materials and Methods; S8 Fig), of the domains, which essentially changes the surfaces in contact with adjacent interstrand repeats. We find that there is only a narrow window of sterically allowed orientations compatible with a 90° bend between sequential spectrin repeats (Fig 6), specifically, a roll between 0° and -60°. The plane of this bend is the same as that which was induced when spectrin was cocrystalized with ankyrin [9]. Further, the BC-loop is peripherally located in this model. This is important because this loop is the insertion site for multiple large domains in spectrins including the SH3 domain (α9; Fig 1A and 1B) [36], calmodulin binding loop (α11; Fig 1B) [37], as well as a PH-domain (tetramerization repeat; Fig 1B) [38–40]. Lastly, this is the same conformation obtained from our cross-link based modeling of the α3-α4 domains in mini-spectrin (Fig 4).

### Hypothetical Mechanism: A Pi-Helical Linker Region

**Fig 7:**
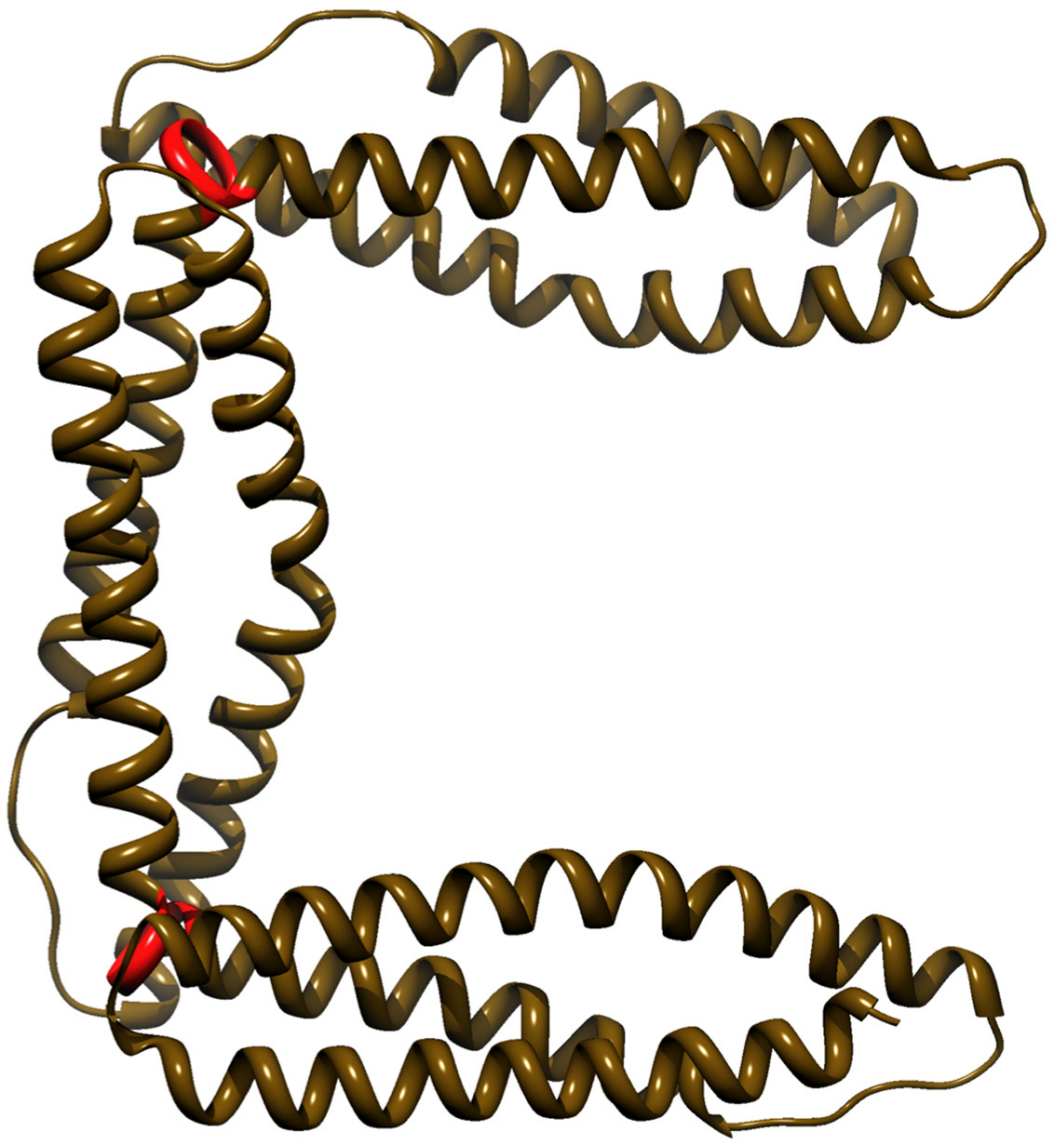
The hypothetical pi-helix. When the linker region (red) is modeled between two consecutive spectrin repeats (brown) that are fixed in accord with the geometry of the Finger Trap model at 60 nm, it assumes a near pi-helical conformation. The missing residues of the linker region were computed and filled in with Modeller 9v1 (29).

It has been established that either extension or contraction of a spring induces a twist in the physical material of the coil. In a spring, this is not typically appreciated because the twist is applied evenly along its length. In contrast, we propose that each spectrin repeat is conformationally invariant to extension-contraction and therefore the twist will be concentrated at the hinge point, which in the Chinese Finger Trap model is the inter-domain linker. Although not absolutely essential for the validity of this model, an elegant way by which spectrin (a right-handed helix) might absorb this twist, while at the same time maintaining tensile integrity, would be if the linker region converts between an approximately alpha helical conformation in the extended form to an approximately pi-helix when contracted. This is indeed, what we find when the linker region is modeled between spectrin repeats in our condensed Chinese Finger Trap Model (Fig 7).

Unlike alpha helices, which are relatively rigid secondary structural elements that can be perpetuated almost indefinitely, pi-helices are short motifs (5–9 residue in length; 1–1.5 turns) typically found near active sites and positions of either allosteric regulation or conformational flexibility [41,42]. The integration of hydrogen bonds into this relatively loosely coordinated structure makes this secondary structural element ideal for permitting significant mobility, while at the same time maintaining structural integrity.

Spectrin’s linker regions (S1 Fig) exhibit substantial sequence similarity to pi-helices found in the protein data bank. Specifically, the primary sequence of pi-helices are characterized by two large hydrophobic residues flanking ~5 generally bulkier amino acids [42] and spectrin’s linker regions are generally composed of five bulky amino acids flanked, primarily, by two highly conserved leucine residues (S1 Fig). Interestingly, the leucine on the N-terminal side of the linker is more conserved than the other flanking leucine, suggesting it is particularly critical. Further, structural analyses of pi-helices present in the protein data bank [42] as well as values obtained from molecular dynamic simulations [43,44] reveal an average phi angle of -76°, a value that prohibits the inclusion of proline into this secondary structural motif. This might serve to explain why five different proline point mutations within linker regions destabilize spectrin and cause hereditary elliptocytoses [45]. That is, the presence of a proline in the linker region is expected to disrupt the spectrin supercoil and could contribute to the disease phenotype.

The presence of this pi-helix might also serve to explain why it has been difficult to characterize the interactions between any two complementary pairs of spectrin repeats on antiparallel strands (i.e. green bars in Fig 1A) and why there is no high-resolution data for any of these interactions. An alpha—to—pi-helix transition changes the rotational orientation of sequential, tandem spectrin repeats. If, two, complementary spectrin repeats on antiparallel strands are laterally associated (e.g., B_5_-A_17_, Fig 1A), in the presence of an alpha helical linker region, the adjacent, tandem complementary pairs (B_6_-A_16_, Fig 1A) would be unable to laterally associate because they would be rotationally misaligned.

## Discussion

### The Chinese Finger Trap Model

In the current study, we sought to reconcile the disparity between spectrin’s physiological resting length with the Extended Model and, in the process, provide a mechanism to explain how spectrin tetramers remain linear over a wide range of molecular lengths. The proposed Chinese Finger Trap Model accomplishes both of these goals and is consistent with all available biochemical and structural data. At shorter lengths, spectrin is a hollow tube that extends by increasing the pitch of each spectrin repeat, thereby decreasing the internal diameter spectrin (Fig 3C), analogous to a Chinese Finger Trap.

The physical dimensions of the spectrin repeat and the geometry of the Chinese Finger Trap Model are consistent with the short ~55–65 nm physiological resting length. The resting position of a tension spring occurs when the sides of each coil are in contact with one another. From the high-resolution X-ray crystal structures of spectrin, it is known that the width of each spectrin repeat is ~2.5 nm. As our Chinese Finger Trap Model has 20 spectrin repeats arranged along its length (Fig 3B), a lower limit for the minimum molecular length of the Compact Model would be ~50 nm plus contributions from the pitch of the helix and the actin binding domains. The observed ~55–65 nm length of spectrin tetramers under physiological conditions strongly argues that sequential coils are in contact with one another. Furthermore, our model recapitulates the ~180 nm length of the Extended Model. Therefore, in addition to reproducing the physical dimensions of spectrin at the different observed molecular lengths, our model also proposes a mechanism for the seamless 3-fold extension experimentally observed of spectrin. In addition to erythrocytic spectrin, the Chinese Finger Trap model should be relevant to some non-erythrocytic spectrins, such as spectrin heterotetramers in the terminal web that are responsible for the hexagonal arrangement of brush border microvilli. Although the model was not formally discussed, we have previously demonstrated that a Chinese Finger Trap conformation of spectrin is of the right dimensions to bridge the gap between adjacent actin microvillar core bundles in the apical cytoplasm where spectrin forms the terminal web [46].

### Consequences of the Model’s Domain Geometry and Symmetry

The junctional complex is composed of several proteins organized around a central actin protofilament of ~14 actin monomers. Similar to the orientation of actin in most if not all cellular structures, the barbed (plus) end of the protofilament is oriented towards the membrane, where it is capped by adducin [47,48] and attached to the membrane through several scaffolding proteins, some of which bind spectrin in addition to actin. At the other (pointed-or- minus) end, actin is capped by tropomodulin. Since all actin filaments exhibit the same polarity, the two actin binding domains on opposite ends of the spectrin heterotetramer that cross-link these filaments must exhibit 2-fold symmetry about a line perpendicular to the long axis of spectrin (and parallel to actin). This attribute, which arises from the antiparallel arrangement of spectrin chains is retained in the supercoiled, Chinese Finger Trap model throughout extension and contraction.

### The “Closed” Heterodimer

The erythroid spectrin heterodimer is able to establish a closed conformation in which the alpha strand loops back to bind its antiparallel partner β-strand and, in the process, form a stable new repeat analogous to the tetramerization repeat [49]. Interestingly, the open-closed dimer equilibrium shifts toward closed dimers at low temperature [50], a condition known to promote extension of spectrin [12]. These data are consistent with the geometry of the Chinese Finger Trap Model acting to limit the formation of closed dimers. Although close in space, the A_0_ and B_17_ domains in the supercoiled heterodimer are antiparallel in orientation (Figs 3C and S5). In order to form a closed heterodimer, the complex must unwind to permit the chains to obtain a parallel orientation required to form the A_0_-B_17_ repeat. This unwinding of the chains would create another barrier to formation of the closed heterodimer: the disparate lengths of alpha (20 repeats) and beta spectrin (16 repeats). Thus, the supercoiled quaternary structure of the Chinese Finger Trap model coupled with the disparate lengths of alpha and beta spectrin might serve to inhibit the formation of the closed spectrin heterodimer, which is physiologically impotent because it is only monovalent for actin. This concept has been directly observed in electron micrographs that depict an uncoiling of the terminal end of a spectrin heterodimer with the formation of either the closed heterodimer or hexameric oligomers [51].

### Erythrocytic, Non-Erythrocytic, and Brain Spectrin

New *in vivo* data suggest that, in certain cell types, brain spectrin appears to be conformationally distinct from erythrocytic as well as some non-erythrocytic spectrin isoforms [20]. When analyzed by sedimentation analysis using sucrose gradients [52,53] as well as rotary shadowed electron microscopy [53–55], brain spectrin was found to be much more rigid. This stiffness (or alternatively an increased predilection towards forming the heterotetramer) could help explain why brain spectrin is not observed to form the “closed” heterodimer [50]. The origins of these divergent properties is unclear from comparing the very similar sequences of these spectrin isoforms (S1 Fig).

### The Restoring Force

*In vivo*, spectrin is believed to act like a spring and is responsible for maintenance of, as well as restoration, of cell shape after mechanical deformation. The Chinese Finger Trap model of spectrin proposes three distinct mechanisms for this restoration force. In the compact conformation (55–65 nm, Fig 3B), our model predicts favorable interactions between adjacent pairs of antiparallel spectrin supercoils (red bars in Fig 3C); however, these interactions are predicted to quickly become irrelevant as spectrin is stretched past 70 nm (Fig 3D) due to the increasing distance between these interfaces. Second, there may exist favorable interactions between sequential spectrin repeats along each strand that are strained as the spectrin tetramer is extended, which will unbend and rotate the linker region, changing the interdomain interfaces between consecutive repeats. A third, and likely most important mechanism for the restoring force derives from interactions between the two complementary anti-parallel strands that we predict exist throughout much of the extension and contraction (green bars in Fig 3C). As the spectrin tetramer is extended and contracted (Fig 3D), the pitch of individual spectrin repeats will change accordingly, which causes the complementary repeats to slide and tilt relative to one another straining their interactions. The precise mechanics of such changes, for example the breaking of interactions between complementary domains vs. the simultaneous distortion of multiple complementary interactions are beyond the scope of this study.

### Unresolved Issues

Although we feel that our Chinese Finger Trap model is of sufficient resolution to predict the overall quaternary structure of the spectrin tetramer, it is not of sufficient resolution to determine the precise interacting surfaces between each complimentary pair of repeats. A significant barrier preventing us from achieving this end is the lack of a high-resolution structure for any pair of spectrin repeats that are predicted to interact with one another, intermolecularly, in either the Extended Model or the Chinese Finger Trap Model. Despite this impediment, a rough approximation of the rotational orientation of each spectrin repeat could be achieved from steric arguments presented in Fig 6. Within these steric constraints of spectrin repeats in each strand, there are technically two different arrangements that can satisfy the two-stranded right handed helical topology of the Chinese Finger Trap model. These two arrangements are schematically represented and compared in S6 Fig. We favor “Mode 1”, also shown in Fig 3, because in this quaternary organization the two antiparallel strands interact via Helices B & C, as opposed to helix A in Mode 2, which is most consistent with published chemical cross-linking data [29–31].

Another unresolved issue is the conformation of alpha 20 through 21 and beta 1 through 2. Unlike other spectrin repeats, these four spectrin repeats are atypical in both their length and linker regions (S1 Fig) and are very similar in sequence and linker length to alpha-actinin, which prompted us, in an earlier study, to create a structural model based on chemical cross-linking and modeling to alpha-actinin [35]. It remains to be determined whether these domains supercoil like we have proposed for the remainder of spectrin or if they are conformationally similar to alpha-actinin. Even if these repeats are not part of the supercoil, it would represent a loss of four repeats from the heterotetramer and the symmetry of the Chinese Finger Trap would remain as described above.

## Materials and Methods

### Modeling the Extended, Crystallographic Structure of the Heterotetramer

The “Crystallographic Structure” of the spectrin heterotetramer was constructed from the crystal structure of alpha spectrin repeats 15–17: PDB Accession ID: 1U4Q [4]. The coordinates of residues 1740–1870 (helix C of the 15^th^ repeat through helix C of the 16^th^ repeat) were excised and the model was constructed by consecutively aligning (C_alpha_) helix C of the 15^th^ repeat (Residues 1740–1764) to helix C of the 16^th^ repeat (Residues 1846–1870); alignment did not include linker regions. A similar approach was published elsewhere [5]. This was repeated until the model structure was 37 repeats long. The second strand was created by rotating a duplicate strand of 37 repeats about the center of mass around a line perpendicular to the long axis.

### Construction of the Chinese Finger Trap

The sequence of all spectrin repeats whose structures have not been solved were modeled onto the crystal structure of the 16^th^ repeat of erythroid alpha spectrin (PDB Accession ID: 1U4Q; [4]) with MODELLER 9v1 [56] in accord with the ClustalW alignment (S1 Fig). While this homology modeling of all repeats was not essential, we wanted to confirm that the variation in repeat lengths by one or two residues did not effect the results. Next, all domains were aligned to one another with the sequence independent, C_alpha_-based algorithm, Topofit [57]. Since, we will propose that the conformation of the linker region in the Chinese Finger Trap model deviates from those present in the x-ray crystal structure, the linker regions were initially omitted.

The electron microscopic analysis of McGough and Josephs [23] assumed that spectrin is a continuous helix (S7 Fig); however, our Chinese Finger Trap model diverges slightly from the former model in that it is composed of a discontinuous square arrangement of linear segments representing individual three helix bundle spectrin repeats (S7 Fig).

The Chinese Finger Trap Model was constructed in several steps, which are schematically represented in S8 Fig. First, the N-terminal C_alpha_ is placed at the origin and each spectrin repeat is oriented in space such that the Z-axis passes through the C_alpha_’s of the two hydrophobic leucines that delimit the repeat. Next a roll is applied, which has the effect of modulating the interdomain interactions in the final model. A pitch is then added which is calculated from the length of spectrin being modeled and can be calculated with eq 1, where P is pitch, length is the length of the spectrin heterotetramer, and 37 is the number of spectrin repeats in one strand. Here, contour length (C_L_) is defined as the end-to-end distance around the helix between two equally phased points and in our model corresponds to 4 spectrin repeats. Next, each spectrin repeat is translated along the x-axis to the proper radius, which is also dependent on the length of spectrin and which can be calculated using eq 4. Finally, each consecutive repeat is rotated about the Z-axis in increasing increments of 90° and translated down the Z-axis by the pitch of each repeat. In an analogous manner to that employed in the extended, “Crystallographic Structure” of spectrin the second strand is created by rotating a duplicate copy about the center of mass about a line perpendicular to the long axis and translating the two strands with respect to one another so that the two strands contact one another. Since the precise interdomain interfaces in contact with one another are not known, this model was created on the premise of avoiding all steric clashes. No energy minimization calculations were employed.

$$P=4•\frac{Length}{37}$$ (1)

$$C_{L}^{2}=P^{2}+{\left(2\pi r\right)}^{2}$$ (2)

$$C_{L}^{2}=P^{2}+{\left(8r\right)}^{2}$$ (3)

$$r=\frac{\sqrt{C_{L}^{2}-P^{2}}}{8}$$ (4)

### Purification and Crosslinking of Mini-Spectrin

A mini-spectrin construct comprising the first five repeats of α-spectrin and the last two repeats of β-spectrin was expressed and purified as previously described [58]. Chemical crosslinking and crosslink identification were performed as previously described [29,30]. Briefly, LC-MS/MS spectra of tryptic digests from mini-spectrin complexes cross-linked with EDC/sulfo-NHS were compared to those from untreated controls to remove background signals and to facilitate targeted acquisition of high-resolution MS/MS spectra. ZXMiner software described in [30] was then used to identify zero-length cross-linked peptides and the specific cross-linked residues for the mini-spectrin complex [29,30].

### Construction of the α3- α4 Linear and 90-Degree Bend Structures

Four existing crystal structures for spectrin-type repeats (PDB: 1CUN, 1U5P, 3FB2, and 1S35) were used as templates for constructing a linear structure for the α3-α4 domains using MODELLER v9.11’s default optimization routine to generate ten alternative models and the one with the lowest average root mean square deviation (RMSD) among all models was selected. To construct the 90-degree bend model, linker region residues were allowed to adopt a non-helical conformation. Distance constraints derived from two observed crosslinks (K307-E431, K365-E442) in this region were included as upper bounds for the distances between alpha-carbon molecules with a mean of 11.0 Å and a standard deviation of 0.1. This small standard deviation allows a smoothed transition from molecules separated by distances that were within the distance constraint (11.0Å) and those that were larger. MODELLER’s performance was improved by changing optimization speed from normal to slow, increasing the allowed number of conjugate gradient iterations to 300, and setting the molecular dynamics refinement speed to very slow. The entire optimization routine was also repeated twice for each modeling attempt. Twenty alternative models were generated and the one with the lowest average RMSD was selected.

### Purification and Electron Microscopy Full-Length Spectrin

Isolation of intact red cell membranes, full-length spectrin dimers and full-length spectrin tetramers were purified as previously described [59]. Grids for electron microscopy (EM) of negatively stained samples were prepared by adsorption of 4–8 μL sample onto glow-discharged, carbon-coated grids, in the absence or presence of 0.25% tannic acid, washed with 300 mM KCl, 30 mM NaCl, 15 mM MgCl_2_, 5 mM HEPES pH 7.4, 0.2 mM DTT, 0.1 mM ATP, 0.02% NaN_3_, and negatively stained with 1% uranyl acetate. For electron cryomicroscopy samples were adsorbed onto Quantifoil ‘holey’ grids, then blotted and frozen-hydrated in a Vitrobot cryoplunger. Images were recorded at 28,000–60,000 x magnification on a Philips CM12 transmission EM (100kV) either on Kodak SO-163 film, and digitized with a Nikon 9000 scanner at 2,000 or 4,000 dots per inch, or directly onto a TVIPS 1024x1024 CCD with a 24 μm pixel size. 29 particles were analyzed with ImageJ [60] software to determine radial density profiles from end-on views of spectrin.

## Supporting Information

S1 Fig Multiple sequence alignments of human alpha and beta spectrin repeats. (PDF)

S2 Fig Structure of the Spectrin Heterotetramer (PDF)

S3 Fig Geometric parameters used to describe spectrin. (PDF)

S4 Fig Annotated MS/MS spectra of the K307-E431 cross-link generated by ZXMiner (PDF)

S5 Fig Cartoon representation of a spectrin heterodimer in a compact supercoiled conformation. (PDF)

S6 Fig Comparison of the two potential antiparallel arrangements of the spectrin heterotetramers consistent with the Chinese Finger Trap model. (PDF)

S7 Fig Comparison of the arrangement of spectrin repeats in the previous model (McGough & Josephs, 1990) to that of the Chinese Finger Trap model. (PDF)

S8 Fig Visual representation of the steps used to position the individual spectrin repeats in the Chinese Finger Trap model. (PDF)

S1 Table Computing a theoretical length of spectrin from cellular abundance of an erythrocytic cytoskeletal protein. (PDF)

## References

[1] Yan Y, Winograd E, Viel A, Cronin T, Harrison SC, Branton D. Crystal structure of the repetitive segments of spectrin. Science. 1993;262: 2027–2030. 8266097 10.1126/science.8266097

[2] Pascual J, Pfuhl M, Walther D, Saraste M, Nilges M. Solution structure of the spectrin repeat: a left-handed antiparallel triple-helical coiled-coil. J Mol Biol. 1997;273: 740–751. 9356261 10.1006/jmbi.1997.1344

[3] Grum VL, Macdonald RI, Mondragon A. Structures of two repeats of spectrin suggest models of flexibility. Cell. 1999;98: 523–535. 10481916 10.1016/s0092-8674(00)81980-7

[4] Kusunoki H, Minasov G, Macdonald RI, Mondragon A. Independent movement, dimerization and stability of tandem repeats of chicken brain alpha-spectrin. J Mol Biol. 2004;344: 495–511. 15522301 10.1016/j.jmb.2004.09.019

[5] Kusunoki H, Macdonald RI, Mondragon A. Structural insights into the stability and flexibility of unusual erythroid spectrin repeats. Structure. 2004;12: 645–656. 15062087 10.1016/j.str.2004.02.022

[6] Davis L, Abdi K, Machius M, Brautigam C, Tomchick DR, Bennett V, et al Localization and Structure of the Ankyrin-binding Site on β2-Spectrin. J Biol Chem. 2009;284: 6982–6987. 10.1074/jbc.M809245200 19098307 PMC2652297

[7] Ipsaro JJ, Huang L, Mondragon A. Structures of the spectrin-ankyrin interaction binding domains. Blood. 2009;113: 5385–5393. 10.1182/blood-2008-10-184358 19141864 PMC2689041

[8] Stabach PR, Simonovic E, Ranieri MA, Aboodi MS, Simonovic M, Morrow JS. The structure of the ankyrin-binding site of β-spectrin reveals how tandem spectrin-repeats generate unique ligand-binding properties. Blood. 2009;113: 5377–5384. 10.1182/blood-2008-10-184291 19168783 PMC2689040

[9] Ipsaro JJ, Mondragon A. Structural basis for spectrin recognition by ankyrin. Blood. 2010;115: 4093–4101. 10.1182/blood-2009-11-255604 20101027 PMC2875089

[10] Ipsaro JJ, Harper SL, Messick TE, Marmorstein R, Mondragon A, Speicher DW. Crystal structure and functional interpretation of the erythrocyte spectrin tetramerization domain complex. Blood. 2010;115: 4843–4852. 10.1182/blood-2010-01-261396 20197550 PMC2890174

[11] Mehboob S, Song Y, Witek M, Long F, Santarsiero BD, Johnson ME, et al Crystal structure of the nonerythrocyte alpha-spectrin tetramerization site reveals differences between erythrocyte and nonerythrocyte spectrin tetramer formation. J Biol Chem. 2010;285, 14572–14584. 10.1074/jbc.M109.080028 20228407 PMC2863205

[12] Vertessy BG, Steck TL. Elasticity of the human red cell membrane skeleton. Effects of temperature and denaturants. Biophys J. 1989;55: 255–262. 2713438 10.1016/S0006-3495(89)82800-0 PMC1330466

[13] Steck TL. The organization of proteins in the human red blood cell membrane. A review. J Cell Biol. 1974;62: 1–19. 4600883 10.1083/jcb.62.1.1 PMC2109190

[14] Nermut MV. Visualization of the "membrane skeleton" in human erythrocytes by freeze-etching. Eur J Cell Biol. 1981;25: 265–271. 7333288

[15] Ursitti JA, Pumplin DW, Wade JB, Bloch RJ. Ultrastructure of the human erythrocyte cytoskeleton and its attachment to the membrane. Cell Motil Cytoskeleton. 1991;19: 227–243. 1934084 10.1002/cm.970190402

[16] Ohno S.An ultrastructural study of the cytoplasmic aspects of erythrocyte membranes by a quick-freezing and deep-etching method. J Anat. 1992;180: 315–320. 1506286 PMC1259678

[17] Nans A, Mohandas N, Stokes DL. Native Ultrastructure of the Red Cell Cytoskeleton by Cryo-Electron Tomography. Biophys J. 2011;101: 2341–2350. 10.1016/j.bpj.2011.09.050 22098732 PMC3218374

[18] Takeuchi M, Miyamoto H, Sako Y, Komizu H, Kusumi A. Structure of the erythrocyte membrane skeleton as observed by atomic force microscopy. Biophys J. 1998;74: 2171–2183. 9591644 10.1016/S0006-3495(98)77926-3 PMC1299560

[19] Swihart AH, Mikrut JM, Ketterson JB, Macdonald RC. Atomic force microscopy of the erythrocyte membrane skeleton. J Microsc. 2001;204: 212–225. 11903798 10.1046/j.1365-2818.2001.00960.x

[20] Xu K, Zhong G, Zhuang X. Actin, spectrin, and associated proteins for a periodic cytoskeleton in axons. Science. 2013;339: 452–456. 10.1126/science.1232251 23239625 PMC3815867

[21] Elgsaeter A, Stokke BT, Mikkelsen A, Branton D. The molecular basis of erythrocyte shape. Science. 1986;234: 1217–1223. 3775380 10.1126/science.3775380

[22] Mirijanian DT, Voth GA. Unique elastic properties of the spectrin tetramer as revealed by multiscale coarse-grained modeling. Proc Natl Acad Sci USA. 2008;105: 1204–1208. 10.1073/pnas.0707500105 18202182 PMC2234116

[23] McGough AM, Josephs R. On the structure of erythrocyte spectrin in partially expanded membrane skeletons. Proc Natl Acad Sci USA. 1990;87: 5208–5212. 2367532 10.1073/pnas.87.13.5208 PMC54291

[24] Hirokawa N, Tilney LG, Fujiwara K, Heuser JE. Organization of actin, myosin, and intermediate filaments in the brush border of intestinal epithelial cells. J Cell Biol. 1982;94: 425–443. 7202010 10.1083/jcb.94.2.425 PMC2112874

[25] Rief M, Gautel M, Oesterhelt F, Fernandez JM, Gaub HE. Reversible unfolding of individual titin immunoglobulin domains by AFM. Science. 1997;276: 1109–1112. 9148804 10.1126/science.276.5315.1109

[26] Law R, Carl P, Harper SL, Dalhaimer P, Speicher DW, Discher DE. Cooperativity in forced unfolding of tandem spectrin repeats. Biophys J. 2003;84: 533–544. 12524305 10.1016/S0006-3495(03)74872-3 PMC1302633

[27] Law R, Harper SL, Speicher DW, Discher DE. Influence of lateral association on forced unfolding of antiparallel spectrin heterodimers. J Biol Chem. 2004;279: 16410–16416. 14761982 10.1074/jbc.M313107200

[28] Pearl M, Fishkind D, Mooseker M, Keene D, Keller T. Studies on the spectrin-like protein from the intestinal brush border, TW 260/240, and characterization of its interaction with the cytoskeleton and actin. J Cell Biol. 1984;98: 66–78. 6538573 10.1083/jcb.98.1.66 PMC2112984

[29] Sriswasdi S, Harper SL, Tang HY, Gallagher PG, Speicher DW. Probing large conformational rearrangements in wild-type and mutant spectrin using structural mass spectroscopy. Proc Natl Acad Sci USA. 2014;111: 1801–1806. 10.1073/pnas.1317620111 24453214 PMC3918770

[30] Sriswasdi S, Harper SL, Tang HY, Speicher DW. Enhanced identification of zero-length chemical cross-link using label free quantitation and high resolution fragment ion spectra. J Proteome Res. 2014;13: 898–914. 10.1021/pr400953w 24369724 PMC3975668

[31] Li D, Harper SL, Tang HY, Maksimova Y, Gallagher PG, Speicher DW. A comprehensive model of the spectrin divalent tetramer region using homology modeling and chemical cross-linking of a mini-spectrin. J Biol Chem. 2010;285: 29535–29545. 10.1074/jbc.M110.145573 20610390 PMC2937985

[32] Begg GE, Harper SL, Morris MB, Speicher DW. Initiation of spectrin dimerization involves complementary electrostatic interactions between paired triple-helical bundles. J Biol Chem. 2000;275: 3279–3287. 10652315 10.1074/jbc.275.5.3279

[33] Harper SL, Begg SE, Speicher DW. Role of terminal nonhomologous domains in initiation of human red cell spectrin dimerization. Biochemistry. 2001;40: 9935–9943. 11502188 10.1021/bi0107795

[34] Li D, Harper SL, Speicher DW. Initiation and propagation of spectrin heterodimer assembly involves distinct energetic processes. Biochemistry. 2007;46: 10585–10594. 17713925 10.1021/bi7007905

[35] Li D, Tang H-Y, Speicher DW. A structural model of the erythrocyte spectrin heterodimer initiation site determined using homology modeling and chemical cross-linking. J Biol Chem. 2008;283: 1553–1562. 17977835 10.1074/jbc.M706981200

[36] Musacchio A, Noble M, Pauptit R, Wierenga R, Saraste M. Crystal structure of a Src-homology 3 (SH3) domain. Nature. 1992;359: 851–855. 1279434 10.1038/359851a0

[37] Simonovic M, Zhang Z, Cianci CD, Steitz TA, Morrow JS. Structure of the calmodulin alphaII-spectrin complex provides insight into the regulation of cell plasticity. J Biol Chem. 2006;281: 34333–34340. 16945920 10.1074/jbc.M604613200

[38] Macias MJ, Musacchio A, Ponstingl H, Nilges M, Saraste M, Oschkinat H. Structure of the pleckstrin homology domain from beta-spectrin. Nature. 1994;369: 675–677. 8208297 10.1038/369675a0

[39] Zhang P, Talluri S, Deng H, Branton D, Wagner G. Solution structure of the pleckstrin homology domain of Drosophila beta-spectrin. Structure. 1995;3: 1185–1195. 8591029 10.1016/s0969-2126(01)00254-4

[40] Nilges M, Macias NJ, O’Donoghue SI, Oschkinat H. Automated NOESY interpretation with ambiguous distance restraints: the refined NMR solution structure of the pleckstrin homology domain from beta-spectrin. J Mol Biol. 1997;269: 408–422. 9199409 10.1006/jmbi.1997.1044

[41] Weaver TM. The pi-helix translates structure into function. Protein Sci. 2000;9: 201–206. 10739264 10.1110/ps.9.1.201 PMC2144447

[42] Fodje MN, Al-Karadaghi S. Occurrence, conformational features and amino acid propensities for the pi-helix. Protein Eng. 2002;15: 353–358. 12034854 10.1093/protein/15.5.353

[43] Lee KH, Benson DR, Kuczera K. Transitions from alpha to pi helix observed in molecular dynamics simulations of synthetic peptides. Biochemistry. 2000;39: 13737–13747. 11076513 10.1021/bi001126b

[44] Armen R, Alonso DO, Daggett V. The role of alpha-, 3(10)-, and pi-helix in helix—>coil transitions. Protein Sci. 2003;12: 1145–1157. 12761385 10.1110/ps.0240103 PMC2323891

[45] Giorgi M, Cianci CD, Gallagher PG, Morrow JS. Spectrin oligomerization is cooperatively coupled to membrane assembly: a linkage targeted by many hereditary hemolytic anemias?. Exp Mol Pathol. 2001;70, 215–230. 11418000 10.1006/exmp.2001.2377

[46] Brown JW, McKnight CJ. Molecular Model of the Microvillus and Organization of the Brush Border. PLoS ONE. 2010;5: e9406 10.1371/journal.pone.0009406 20195380 PMC2827561

[47] Kuhlman PA, Hughes CA, Bennett V, Fowler VM. A new function for adducin. Calcium/calmodulin-regulated capping of the barbed ends of actin filaments. J Biol Chem. 1996;271: 7986–7991. 8626479 10.1074/jbc.271.14.7986

[48] Kuhlman PA, Fowler VM. Purification and characterization of an alpha 1 beta 2 isoform of CapZ from human erythrocytes: cytosolic location and inability to bind to Mg2+ ghosts suggest that erythrocyte actin filaments are capped by adducin. Biochemistry. 1997;36: 13461–13472. 9354614 10.1021/bi970601b

[49] Speicher DW, DeSilva TM, Speicher KD, Ursitti JA, Hembach P, Weglarz L. Location of the human red cell spectrin tetramer binding site and detection of a related "closed" hairpin loop dimer using proteolytic footprinting. J Biol Chem. 1993;268: 4227–4235. 8440706

[50] Begg GE, Morris MB, Ralston GB. Comparison of the salt-dependent self-association of brain and erythroid spectrin. Biochemistry. 1997;36: 6977–6985. 9188694 10.1021/bi970186n

[51] Morrow JS, Marchesi VT. Self-assembly of spectrin oligomers in vitro: a basis for a dynamic cytoskeleton. J Cell Bio. 1981;l 88: 463–468. 7204503 10.1083/jcb.88.2.463 PMC2111738

[52] Burridge K, Kelly T, Mangeat P. Nonerythrocytic spectrins: actin-membrane attachment proteins occurring in many cell types. J Cell Biol. 1982;95: 478–486. 6183274 10.1083/jcb.95.2.478 PMC2112974

[53] Davis J, Bennett V. Brain spectrin—isolation of subunits and formation of hybrids with erythrocytic spectrin subunits. J Biol Chem. 1983;258: 7757–7766. 6863263

[54] Bennett V, Davis J, Fowler WE. Brain spectrin, a membrane-associated protein related in structure and function to erythrocytic spectrin. Nature. 1982;299: 126–131. 7110333 10.1038/299126a0

[55] Glenney JR, Glenney P, Osborn M, Weber K. An F-Actin- and calmodulin binding protein from isolated intestinal brush borders has a morphology related to spectrin. Cell. 1982;28: 843–854. 7201352 10.1016/0092-8674(82)90063-0

[56] Sali A, Blundell TL. Comparative protein modeling by satisfaction of spatial restraints. J Mol Biol. 1993;234: 779–815. 8254673 10.1006/jmbi.1993.1626

[57] Ilyin VA, Abyzov A, Leslin CM. Structural alignment of proteins by a novel TOPOFIT method, as a superimposition of common volumes at a topomax point. Protein Sci. 2004;13: 1865–1874. 15215530 10.1110/ps.04672604 PMC2279929

[58] Harper SL, Li D, Maksimova Y, Gallagher PG, Speicher DW. A fused alpha-beta “mini-spectrin” mimics intact erythrocyte spectrin head-to-head tetramer. J Biol Chem. 2010;285: 11003–11012. 10.1074/jbc.M109.083048 20139081 PMC2856305

[59] Speicher DW, Weglarz L, DeSilva TM. Properties of human red cell spectrin heterodimer (side-to-side) assembly and identification of an essential nucleation site. J Biol Chem. 1992;267: 14475–14482. 1634521

[60] Rasband WS. ImageJ, U.S. National Institutes of Health, Bethesda, Maryland, USA, http://imagej.nih.gov/ij/, 1997–2014.

[61] Fairbanks G, Steck TL, Wallach DF. Electrophoretic Analysis of the Major Polypeptides of the Human Erythrocyte Membrane. Biochemistry. 1971;10: 2607–2617. 10.1021/bi00789a030 4326772

[62] Pinder JC, Gratzer WB. Structural and dynamic states of actin in the erythrocyte. J Cell Biol. 1983;96: 768–775. 6682109 10.1083/jcb.96.3.768 PMC2112381

[63] Gardner K, Bennett V. A New Erythrocyte Membrane-Associated Protein with Calmodulin Binding Activity. Identification and Purification. J Biol Chem. 1986;261: 1339–48. 3511042

[64] Bennett V. Spectrin-Based Membrane Skeleton: A Multipotential Adaptor Between Plasma Membrane and Cytoskeleton. Physiol Rev. 1990;70: 1029–1065. 2271059 10.1152/physrev.1990.70.4.1029

[65] Savvides P, Shalev O, John KM, Lux SE. Combined Spectrin and Ankyrin Deficiency is Common in Autosomal Dominant Hereditary Spherocytosis. Blood. 1993;82: 2953–2960. 8219186

[66] Husain-Chishti A, Levin A, Branton D. Abolition of Actin-Bundling by Phosphorylation of Human Erythrocyte Protein 4.9. Nature. 1988;344: 718–721. 2842686 10.1038/334718a0

[67] Shelton RL, Langdon RG. Quantitation of the Major Proteins of the Human Erythrocyte Membrane by Amino Acid Analysis. Anal Biochem. 1984;140: 366–371. 6486423 10.1016/0003-2697(84)90179-9

[68] Fowler VM, Bennett V. Erythrocyte Membrane Tropomyosin. Purification and Properties. J Biol Chem. 1984;259: 5978–5989. 6715382

[69] Fowler VM. Identification and Purification of a Novel Mr 43,000 Tropomyosin-Binding Protein from Human Erythrocyte Membranes. J Biol Chem. 1987;262: 12792–12800. 3624279

