# Supplementary material for: The Physiological Molecular Shape of Spectrin: A Compact Supercoil Resembling a Chinese Finger Trap
Source: PLoS Comput Biol. 2015 Jun 11;11(6):e1004302. doi: 10.1371/journal.pcbi.1004302 (PMC4466138; doi:10.1371/journal.pcbi.1004302)
Supplement: S1 Fig — (PDF) [file pcbi.1004302.s001.pdf]

```
>SPTA1_A0      ------------------------------------------------------------------SGP----KV-LETAEEIQERRQEVLTRYQSFKERVAERGQKL    EDSYH-------------
>SPTA1_A1      LQVFKRDADDLGKWIMEKVNILT--DKSYEDPTNIQGKYQKHQSLEAEVQTKSRL-MSELEKTREERFTMG--HS-AH--EETKAHIEELRHLWDLLLELTLEKGDQL    LRALK-------------
>SPTA1_A2      FQQYVQECADILEWIGDKEAIAT-SVELGEDWERTEVLHKKFEDFQVELVAK-EGRVVEVNQYANECAEEN-----HPDLPLIQSKQNEVNAAWERLRGLALQRQKAL    SNAAN-------------
>SPTA1_A3      LQRFKRDVTEAIQWIKEKEPVLT-SEDYGKDLVASEGLFHSHKGLERNLAVMSDK-VKELCAKAEKLTL---SHP--SDAPQIQEMKEDLVSSWEHIRALATSRYEKL    QATYW-------------
>SPTA1_A4      YHRFSSDFDELSGWMNEKTAAIN-ADELPTDVAGGEVLLDRHQQHKHEIDSYDDR-FQSADETGQDLVNAN---H-EAS-DEVREKMEILDNNWTALLELWDERHRQY    EQCLD-------------
>SPTA1_A5      FHLFYRDSEQVDSWMSRQEAFLE-NEDLGNSLGSAEALLQKHEDFEEAFTAQEEK-IITVDKTATKLIG-DDHYD-SEN---IKAIRDGLLARRDALREKAATRRRLL    KESLL-------------
>SPTA1_A6      LQKLYEDSDDLKNWINKKKKLAD--DEDYKDIQNLKSRVQKQQVFEKELAVNKTQ-LENIQKTGQEMIEGG--HY-AS--DNVTTRLSEVASLWEELLEATKQKGTQL    HEANQ-------------
>SPTA1_A7      QLQFENNAEDLQRWLEDVEWQVT-SEDYGKGLAEVQNRLRKHGLLESAVAARQDQ-VDILTDLAAYFEEIG-----HPDSKDIRARQESLVCRFEALKEPLATRKKKL    LDLLH-------------
>SPTA1_A8      LQLICRDTEDEEAWIQETEPSAT-STYLGKDLIASKKLLNRHRVILENIASHEPRIQEITERGNK-MVE----EG-HFAAEDVASRVKSLNQNMESLRARAARRQNDL    EANVQ-------------
>SPTA1_A9-10   FQQYLADLHEAETWIREKEPIVD-NTNYGADEEAAGALLKKHEAFLLDLNSFGDS-MKALRNQANAC-----------------------------------------    ------------------
**Omitted the SH3 domain**
>SPTA1_A9-10   ------------------------------------------------------------------------------PGN--ITQRQEQIENQYRSLLDRAEERRRL    LQRYN-------------
>SPTA1_A11     EFLLAYEAGDMLEWIQEKKAENT-GVEL----DDVWELQKKFDEFQKDLNTN-EPRLRDINKVADDLLFEG-----LLTP-EGAQIRQELNSRWGSLQRLADEQRLL    GSAHA-------------
>SPTA1_A12     VEVFHREADDTKEQIEKKCQALS-AADPGSDLFSVQALQRRHEGFERDLVPLGDK-VTILGETAERLSE---SHP--DATEDLQRQKMELNEAWEDLQGRTKDRKESL    NEAQK-------------
>SPTA1_A13     FYLFLSKARDLQNWISSIGGMVS-SQELAEDLTGIEILLERHQEHRADMEAEAPT-FQALEDFSAELIDSG---H-HAS-PEIEKKLQAVKLERDDLEKAWEKRKKIL    DQCLE-------------
>SPTA1_A14     LQMFQGNCDQVESWMVARENSLR-SDDK-SSLDSLEALMKKRDDLDKAITAQEGK-ITDLEHFAESLIA-DEHYA-KEE---IATRLQRVLDRWKALKAQLIDERTKL    GDYAN-------------
>SPTA1_A15     LKQFYRDLEELEEWISEMLPTAC--DESYKDATNIQRKYLKHQTFAHEVDGRSEQ-VHGVINLGNSLIECS--AC-DGNEEAMKEQLEQLKEHWDHLLERTNDKGKKL    NEASR-------------
>SPTA1_A16     QQRFNTSIRDFEFWLSEAETLLA-MKDQARDLASAGNLLKKHQLLEREMLAREDA-LKDLNTLAEDLLSSG-----TFNVDQIVKKKDNVNKRFLNVQELAAAHHEKL    KEAYA-------------
>SPTA1_A17     LFQFFQDLDDEESWIEEKLIRVS-SQDYGRDLQGVQNLLKKHKRLEGELVAHEPAIQNVLDMAEK-LKD----KA-AVGQEEIQLRLAQFVEHWEKLKELAKARGLKL    EESLE-------------
>SPTA1_A18     YLQFMQNAEEEEAWINEKNALAV-RGDCGDTLAATQSLLMKHEALENDFAVHETR-VQNVCAQGEDILN-KVLQE-ESQNKEISSKIEALNEKTPSLAKAIAAWKLQL    EDDYA-------------
>SPTA1_A19     FQEFNWKADVVEAWIADKETSLK-TNGNGADLGDFLTLLAKQDTLDASLQSFQQERLPEITDLKDKLISAQ-----HNQSKAIEERYAALLKRWEQLLEASAVHRQKL    LEKQLPLQKAEDL-----
>SPTA1_A20     FVEFAHKASALNNWCEKMEENLS-EPVHCVSLNEIRQLQKDHEDFLASLARAQADFK-----CLLELDQQIKALG-VPSSPYTWLTVEVLERTWKHLSDIIEEREQEL    QKEEARQVKNFE------
>SPTA1_A21     CQEFEQNASTFLQWILETRAYFLDGS-LLKETGTLESQLEANKRKQKEIQAMKRQ-LTKIVDLGDNLEDAL-----ILDIKY---STIGLAQQWDQLYQLGLRMQHNL    EQQIQAKDIKGVSEETLK

>SPTA2_A0      ------------------------------------------------------------------SGV----KV-LETAEDIQERRQQVLDRYHRFKELSTLRRQKL    EDSYR-------------
>SPTA2_A1      FQFFQRDAEELEKWIQEKLQIAS--DENYKDPTNLQGKLQKHQAFEAEVQANSGA-IVKLDETGNLMISEG--HF-AS--ETIRTRLMELHRQWELLLEKMREKGIKL    LQAQK-------------
>SPTA2_A2      LVQYLRECEDVMDWINDKEAIVT-SEELGQDLEHVEVLQKKFEEFQTDMAAH-EERVNEVNQFAAKLIQEQ-----HPEEELIKTKQDEVNAAWQRLKGLALQRQGKL    FGAAE-------------
>SPTA2_A3      VQRFNRDVDETISWIKEKEQLMA-SDDFGRDLASVQALLRKHEGLERDLAALEDK-VKALCAEADRLQQ---SHP--LSATQIQVKREELITNWEQIRTLAAERHARL    NDSYR-------------
>SPTA2_A4      LQRFLADFRDLTSWVTEMKALIN-ADELASDVAGAEALLDRHQEHKGEIDAHEDS-FKSADESGQALLAAG---H-YAS-DEVREKLTVLSEERAALLELWELRRQQY    EQCMD-------------
>SPTA2_A5      LQLFYRDTEQVDNWMSKQEAFLL-NEDLGDSLDSVEALLKKHEDFEKSLSAQEEK-ITALDEFATKLIQ-NNHYA-MED---VATRRDALLSRRNALHERAMRRRAQL    ADSFH-------------
>SPTA2_A6      LQQFFRDSDELKSWVNEKMKTAT--DEAYKDPSNLQGKVQKHQAFEAELSANQSR-IDALEKAGQKLIDVN--HY-AK--DEVAARMNEVISLWKKLLEATELKGIKL    REANQ-------------
>SPTA2_A7      QQQFNRNVEDIELWLYEVEGHLA-SDDYGKDLTNVQNLQKKHALLEADVAAHQDR-IDGITIQARQFQDAG-----HFDAENIKKKQEALVARYEALKEPMVARKQKL    ADSLR-------------
>SPTA2_A8      LQQLFRDVEDEETWIREKEPIAA-STNRGKDLIGVQNLLKKHQALQAEIAGHEPRIKAVTQKGNA-MVE----EG-HFAAEDVKAKLHELNQKWEALKAKASQRRQDL    EDSLQ-------------
>SPTA2_A9-10   AQQYFADANEAESWMREKEPIVG-STDYGKDEDSAEALLKKHEALMSDLSAYGSS-IQALREQAQSCR----------------------------------------    ------------------
**Omitted the SH3 domain**
>SPTA2_A9_10   ----------------------------------------------------------------------------------VSLRMKQVEELYHSLLELGEKRKGML    EKSCK-------------
>SPTA2_A11     KFMLFREANELQQWINEKEAALT-SEEVGADLEQVEVLQKKFDDFQKDLKAN-ESRLKDINKVAEDLESEG-----LMAEEVQAVQQQEVY-----------------    ------------------
**Omitted the calmodulin binding loop**
>SPTA2_A11     --------------------------------------------------------------------FN--------------SIK-ELNERWRSLQQLAEERSQLL    GSAHE-------------
>SPTA2_A12     VQRFHRDADETKEWIEEKNQALN-TDNYGHDLASVQALQRKHEGFERDLAALGDK-VNSLGETAERLIQ---SHP--ESAEDLQEKCTELNQAWSSLGKRADQRKAKL    GDSHD-------------
>SPTA2_A13     LQRFLSDFRDLMSWINGIRGLVS-SDELAKDVTGAEALLERHQEHRTEIDARAGT-FQAFEQFGQQLLAHG---H-YAS-PEIKQKLDILDQERADLEKAWVQRRMML    DQCLE-------------
>SPTA2_A14     LQLFHRDCEQAENWMAAREAFLN-TEDKGDSLDSVEALIKKHEDFDKAINVQEEK-IAALQAFADQLIA-AGHYA-KGD---ISSRRNEVLDRWRRLKAQMIEKRSKL    GESQT-------------
>SPTA2_A15     LQQFSRDVDEIEAWISEKLQTAS--DESYKDPTNIQSKHQKHQAFEAELHANADR-IRGVIDMGNSLIERG--AC-AGSEDAVKARLAALADQWQFLVQKSAEKSQKL    KEANK-------------
>SPTA2_A16     QQNFNTGIKDFDFWLSEVEALLA-SEDYGKDLASVNNLLKKHQLLEADISAHEDR-LKDLNSQADSLMTSS-----AFDTSQVKDKRDTINGRFQKIKSMAASRRAKL    NESHR-------------
>SPTA2_A17     LHQFFRDMDDEESWIKEKKLLVG-SEDYGRDLTGVQNLRKKHKRLEAELAAHEPAIQGVLDTGKK-LSD----DN-TIGKEEIQQRLAQFVEHWKELKQLAAARGQRL    EESLE-------------
>SPTA2_A18     YQQFVANVEEEEAWINEKMTLVA-SEDYGDTLAAIQGLLKKHEAFETDFTVHKDR-VNDVCTNGQDLIK-KNNHH-EEN---ISSKMKGLNGKVSDLEKAAAQRKAKL    DENSA-------------
>SPTA2_A19     FLQFNWKADVVESWIGEKENSLK-TDDYGRDLSSVQTLLTKQETFDAGLQAFQQEGIANITALKDQLLAAK-----HVQSKAIEARHASLMKRWSQLLANSAARKKKL    LEAQSHFRKVEDL-----
>SPTA2_A20     FLTFAKKASAFNSWFENAEEDLT-DPVRCNSLEEIKALREAHDAFRSSLSSAQADFN-----QLAELDRQIKSFR-VASNPYTWFTMEALEETWRNLQKIIKERELEL    QKEQRRQEENDK------
>SPTA2_A21     RQEFAQHANAFHQWIQETRTYLLDGSCMVEESGTLESQLEATKRKHQEIRAMRSQ-LKKIEDLGAAMEEAL-----ILDNKYTEHSTVGLAQQWDQLDQLGMRMQHNL    EQQIQARNTTGVTEEALK
```

Supplemental Figure 1A *Multiple sequence alignments of human alpha spectrin repeats.* The linker regions between sequential spectrin repeats are separated to the right. Accessory domains that exist within spectrin were omitted.

```
>SPTB1_B1     IEKYSGLASDLLTWIEQTITVLN-SRKFANSLTGVQQQLQAFSTYRTVEKPPKFQEKGNLEVLLFTIQSRMRANNQKVYTPHDGKLVSDINRAWESLEEAEYRRELAL   RNELIRQEKLEQL-----
>SPTB1_B2     ARRFDRKAAMRETWLSENQRLVA-QDNFGYDLAAVEAAKKKHEAIETDTAAYEER-VRALEDLAQELEK---ENY--HDQKRITARKDNILRLWSYLQELLQSRRQRL   ETTLA-------------
>SPTB1_B3     LQKLFQDMLHSIDWMDEIKAHLL-SAEFGKHLLEVEDLLQKHKLMEADIAIQGDK-VKAITAATLKFTE-GKGYQ-PCDPQVIQDRISHLEQCFEELSNMAAGRKAQL   EQSKR-------------
>SPTB1_B4     LWKFFWEMDEAESWIKEKEQIYS-SLDYGKDLTSVLILQRKHKAFEDELRGLDAHLEQIFQEAHG-MVA----RK-QFGHPQIEARIKEVSAQWDQLKDLAAFCKKNL   QDAEN-------------
>SPTB1_B5     FFQFQGDADDLKAWLQDAHRLLS-GEDVGQDEGATRALGKKHKDFLEELEESRGV-MEHLEQQAQGFP--EEFRD-SPD---VTHRLQALRELYQQVVAQADLRQQRL   QEALD-------------
>SPTB1_B6     LYTVFGETDACELWMGEKEKWLA-EMEMPDTLEDLEVVQHRFDILDQEMKTL-MTQIDGVNLAANSLVESG-----HPRSREVKQYQDHLNTRWQAFQTLVSERREAV   DSALR-------------
>SPTB1_B7     VHNYCVDCEETSKWITDKTKVVESTKDLGRDLAGIIAIQRKLSGLERDVAAIQAR-VDALERESQQLMD---SHP--EQKEDIGQRQKHLEELWQGLQQSLQGQEDLL   GEVSQ-------------
>SPTB1_B8     LQAFLQDLDDFQAWLSITQKAVA-SEDMPESLPEAEQLLQQHAGIKDEIDGHQDS-YQRVKESGEKVIQGQ---T-DPEYLLLGQRLEGLDTGWNALGRMWESRSHTL   AQCLG-------------
>SPTB1_B9     FQEFQKDAKQAEAILSNQEYTLA-HLEPPDSLEAAEAGIRKFEDFLGSMENNRDK-VLSPVDSGNKLVA-EGNLY-SDK---IKEKVQLIEDRHRKNNEKAQEASVLL   RDNLE-------------
>SPTB1_B10    LQNFLQNCQELTLWINDKLLTSQ--DVSYDEARNLHNKWLKHQAFVAELASHEGW-LENIDAEGKQLMDEK--PQ-FT--ALVSQKLEALHRLWDELQATTKEKTQHL   SAARS-------------
>SPTB1_B11    SDLRLQTHADLNKWISAMEDQLR-SDDPGKDLTSVNRMLAKLKRVEDQVNVRKEE-LGELFAQVPSMGEE------GGDAD------LSIEKRFLDLLEPLGRRKKQL   ESSRA-------------
>SPTB1_B12    KLQISRDLEDETLWVEERLPLAQ-SADYGTNLQTVQLFMKKNQTLQNEILGHTPRVEDVLQRGQQ-LVE----AA-EIDCQDLEERLGHLQSSWDRLREAAAGRLQRL   RDANE-------------
>SPTB1_B13    AQQYYLDADEAEAWIGEQELYVI-SDEIPKDEEGAIVMLKRHLRQQRAVEDYGRN-IKQLASRAQGLLS----AG-HPEGEQIIRLQGQVDKHYAGLKDVAEERKRKL   ENMYH-------------
>SPTB1_B14    LFQLKRETDDLEQWISEKELVAS-SPEMGQDFDHVTLLRDKFRDFARETGAIGQERVDNVNAFIERLIDAG-----HSEAATIAEWKDGLNEMWADLLELIDTRMQLL   AASYD-------------
>SPTB1_B15    LHRYFYTGAEILGLIDEKHRELP--EDVGLDASTAESFHRVHTAFERELHLLGVQ-VQQFQDVATRLQT---AYA-GEKAEAIQNKEQEVSAAWQALLDACAGRRTQL   VDTAD-------------
>SPTB1_B16    KFRFFSMARDLLSWMESIIRQIE-TQERPRDVSSVELLMKYHQGINAEIETRSKN-FSACLELGESLLQRQ---H-QAS-EEIREKLQQVMSRRKEMNEKWEARWERL   RMLLE-------------
>SPTB1_B17    VCQFSRDASVAEAWLIAQEPYLA-SGDFGHTVDSVEKLIKRHEAFEKSTASWAER-FAAL------------------------------------------------   ------------------

>SPTB2_B1     IEKYESLASDLLEWIEQTIIILN-NRKFANSLVGVQQQLQAFNTYRTVEKPPKFTEKGNLEVLLFTIQSKMRANNQKVYMPREGKLISDINKAWERLEKAEHERELAL   RNELIRQEKLEQL-----
>SPTB2_B2     ARRFDRKAAMRETWLSENQRLVS-QDNFGFDLPAVEAATKKHEAIETDIAAYEER-VQAVVAVARELEA---ENY--HDIKRITARKDNVIRLWEYLLELLRARRQRL   EMNLG-------------
>SPTB2_B3     LQKIFQEMLYIMDWMDEMKVLVL-SQDYGKHLLGVEDLLQKHTLVEADIGIQAER-VRGVNASAQKFATDGEGYK-PCDPQVIRDRVAHMEFCYQELCQLAAERRARL   EESRR-------------
>SPTB2_B4     LWKFFWEMAEEEGWIREKEKILS-SDDYGKDLTSVMRLLSKHRAFEDEMSGRSGHFEQAIKEGED-MIA----EE-HFGSEKIRERIIYIREQWANLEQLSAIRKKRL   EEASL-------------
>SPTB2_B5     LHQFQADADDIDAWMLDILKIVS-SSDVGHDEYSTQSLVKKHKDVAEEIANYRPT-LDTLHEQASALP--QEHAE-SPD---VRGRLSGIEERYKEVAELTRLRKQAL   QDTLA-------------
>SPTB2_B6     LYKMFSEADACELWIDEKEQWLN-NMQIPEKLEDLEVIQHRFESLEPEMNNQ-ASRVAVVNQIARQLMHSG-----HPSEKEIKAQQDKLNTRWSQFRELVDRKKDAL   LSALS-------------
>SPTB2_B7     IQNYHLECNETKSWIREKTKVIESTQDLGNDLAGVMALQRKLTGMERDLVAIEAK-LSDLQKEAEKLES---EHP--DQAQAILSRLAEISDVWEEMKTTLKNREASL   GEASK-------------
>SPTB2_B8     LQQFLRDLDDFQSWLSRTQTAIA-SEDMPNTLTEAEKLLTQHENIKNEIDNYEED-YQKMRDMGEMVTQGQ---T-DAQYMFLRQRLQALDTGWNELHKMWENRQNLL   SQSHA-------------
>SPTB2_B9     YQQFLRDTKQAEAFLNNQEYVLA-HTEMPTTLEGAEAAIKKQEDFMTTMDANEEK-INAVVETGRRLVS-DGNIN-SDR---IQEKVDSIDDRHRKNRETASELLMRL   KDNRD-------------
>SPTB2_B10    LQKFLQDCQELSLWINEKMLTAQ--DMSYDEARNLHSKWLKHQAFMAELASNKEW-LDKIEKEGMQLISEK--PE-TE--AVVKEKLTGLHKMWEVLESTTQTKAQRL   FDANK-------------
>SPTB2_B11    AELFTQSCADLDKWLHGLESQIQ-SDDYGKDLTSVNILLKKQQMLENQMEVRKKE-IEELQSQAQALSQE------GKSTDEVDSKRLTVQTKFMELLEPLNERKHNL   LASKE-------------
>SPTB2_B12    IHQFNRDVEDEILWVGERMPLAT-STDHGHNLQTVQLLIKKNQTLQKEIQGHQPRIDDIFERSQNIVTD----SS-SLSAEAIRQRLADLKQLWGLLIEETEKRHRRL   EEAHR-------------
>SPTB2_B13    AQQYYFDAAEAEAWMSEQELYMM-SEEKAKDEQSAVSMLKKHQILEQAVEDYAET-VHQLSKTSRALVA----DS-HPESERISMRQSKVDKLYAGLKDLAEERRGKL   DERHR-------------
>SPTB2_B14    LFQLNREVDDLEQWIAEREVVAG-SHELGQDYEHVTMLQERFREFARDTGNIGQERVDTVNHLADELINSG-----HSDAATIAEWKDGLNEAWADLLELIDTRTQIL   AASYE-------------
>SPTB2_B15    LHKFYHDAKEIFGRIQDKHKKLP--EELGRDQNTVETLQRMHTTFEHDIQALGTQ-VRQLQEDAARLQA---AYA-GDKADDIQKRENEVLEAWKSLLDACESRRVRL   VDTGD-------------
>SPTB2_B16    KFRFFSMVRDLMLWMEDVIRQIE-AQEKPRDVSSVELLMNNHQGIKAEIDARNDS-FTTCIELGKSLLARK---H-YAS-EEIKEKLLQLTEKRKEMIDKWEDRWEWL   RLILE-------------
>SPTB2_B17    VHQFSRDASVAEAWLLGQEPYLS-SREIGQSVDEVEKLIKRHEAFEKSAATWDE------------------------------------------------------   ------------------

>SPTN2_B1     VEKYESLASELLQWIEQTIVTLN-DRQLANSLSGVQNQLQSFNSYRTVEKPPKFTEKGNLEVLLFTIQSKLRANNQKVYTPREGRLISDINKAWERLEKAEHERELAL   RTELIRQEKLEQL-----
>SPTN2_B2     AARFDRKAAMRETWLSENQRLVS-QDNFGLELAAVEAAVRKHEAIETDIVAYSGR-VQAVDAVAAELAA---ERY--HDIKRIAARQHNVARLWDFLRQMVAARRERL   LLNLE-------------
>SPTN2_B3     LQKVFQDLLYLMDWMEEMKGRLQ-SQDLGRHLAGVEDLLQLHELVEADIAVQAER-VRAVSASALRFCNPGKEYR-PCDPQLVSERVAKLEQSYEALCELAAARRARL   EESRR-------------
>SPTN2_B4     LWRFLWEVGEAEAWVREQQHLLA-SADTGRDLTGALRLLNKHTALRGEMSGRLGPLKLTLEQGQQ-LVA----EG-HPGASQASARAAELQAQWERLEALAEERAQRL   AQAAS-------------
>SPTN2_B5     LYQFQADANDMEAWLVDALRLVS-SPELGHDEFSTQALARQHRALEEEIRSHRPT-LDALREQAAALP--PTLSR-TPE---VQSRVPTLERHYEELQARAGERARAL   EAALA-------------
>SPTN2_B6     LYTMLSEAGACGLWVEEKEQWLN-GLALPERLEDLEVVQQRFETLEPEMNTL-AAQITAVNDIAEQLLKAN-----PPGKDRIVNTQEQLNHRWQQFRRLADGKKAAL   TSALS-------------
>SPTN2_B7     IQNYHLECTETQAWMREKTKVIESTQGLGNDLAGVLALQRKLAGTERDLEAIAAR-VGELTREANALAA---GHP--AQAVAINARLREVQTGWEDLRATMRRREESL   GEARR-------------
>SPTN2_B8     LQDFLRSLDDFQAWLGRTQTAVA-SEEGPATLPEAEALLAQHAALRGEVERAQSE-YSRLRALGEEVTRDQ---A-DPQCLFLRQRLEALGTGWEELGRMWESRQGRL   AQAHG-------------
>SPTN2_B9     FQGFLRDARQAEGVLSSQEYVLS-HTEMPGTLQAADAAIKKLEDFMSTMDANGER-IHGLLEAGRQLVS-EGNIH-ADK---IREKADSIERRHKKNQDAAQQFLGRL   RDNRE-------------
>SPTN2_B10    QQHFLQDCHELKLWIDEKMLTAQ--DVSYDEARNLHTKWQKHQAFMAELAANKDW-LDKVDKEGRELTLEK--PE-LK--ALVSEKLRDLHRRWDELETTTQAKARSL   FDANR-------------
>SPTN2_B11    AELFAQSCCALESWLESLQAQLH-SDDYGKDLTSVNILLKKQQMLEWEMAVREKE-VEAIQAQAKALAQE------DQGAGEVERTSRAVEEKFRALCQPMRERCRRL   QASRE-------------
>SPTN2_B12    QHQFHRDVEDEILWVTERLPMAS-SMEHGKDLPSVQLLMKKNQTLQKEIQGHEPRIADLRERQR------------ALGAAAAGPELAELQEMWKRLGHELELRGKRL   EDALR-------------
>SPTN2_B13    AQQFYRDAAEAEAWMGEQELHMM-GQEKAKDELSAQAEVKKHQVLEQALADYAQT-IHQLAASSQDMID----HE-HPESTRISIRQAQVDKLYAGLKELAGERRERL   QEHLR-------------
>SPTN2_B14    LCQLRRELDDLEQWIQEREVVAA-SHELGQDYEHVTMLRDKFREFSRDTSTIGQERVDSANALANGLIAGG-----HAARATVAEWKDSLNEAWADLLELLDTRGQVL   AAAYE-------------
>SPTN2_B15    LQRFLHGARQALARVQHKQQQLP--DGTGRDLNAAEALQRRHCAYEHDIQALSPQ-VQQVQDDGHRLQK---AYA-GDKAEEIGRHMQAVAEAWAQLQGSSAARRQLL   LDTTD-------------
>SPTN2_B16    KFRFFKAVRELMLWMDEVNLQMD-AQERPRDVSSADLVIKNQQGIKAEIEARADR-FSSCIDMGKELLARS---H-YAA-EEISEKLSQLQARRQETAEKWQEKMDWL   QLVLE-------------
>SPTN2_B17    VLVFGRDAGMAEAWLCSQEPLVR-SAELGCTVDEVESLIKRHEAFQKSAVAWEER-FCAL------------------------------------------------   ------------------
```

Supplemental Figure 1B *Multiple sequence alignments of human beta spectrin repeats.* The linker regions between sequential spectrin repeats are separated to the right. Accessory domains that exist within spectrin were omitted.
