# Supplementary material for: The Physiological Molecular Shape of Spectrin: A Compact Supercoil Resembling a Chinese Finger Trap
Source: PLoS Comput Biol. 2015 Jun 11;11(6):e1004302. doi: 10.1371/journal.pcbi.1004302 (PMC4466138; doi:10.1371/journal.pcbi.1004302)
Supplement: S2 Fig — (PDF) [file pcbi.1004302.s002.pdf]

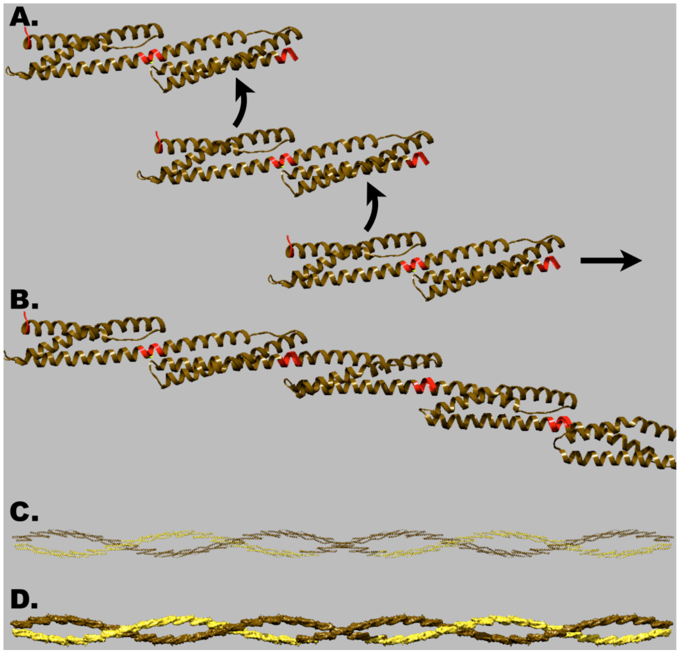

<u>Supplemental Figure 2</u> *Crystallographic Structure of the Spectrin Heterotetramer***. A, B**. Schematic representation of sequentially concatenating spectrin di-repeats to create a continuous model of 37 spectrin repeats. **C**. Ribbon diagram of the crystallographic spectrin heterotetramer. **D**. Surface representation of the crystallographic spectrin heterotetramer. Alpha-spectrin is colored brown, beta-spectrin yellow, and the linker region red.
