# Supplementary material for: The Physiological Molecular Shape of Spectrin: A Compact Supercoil Resembling a Chinese Finger Trap
Source: PLoS Comput Biol. 2015 Jun 11;11(6):e1004302. doi: 10.1371/journal.pcbi.1004302 (PMC4466138; doi:10.1371/journal.pcbi.1004302)
Supplement: S3 Fig — (PDF) [file pcbi.1004302.s003.pdf]

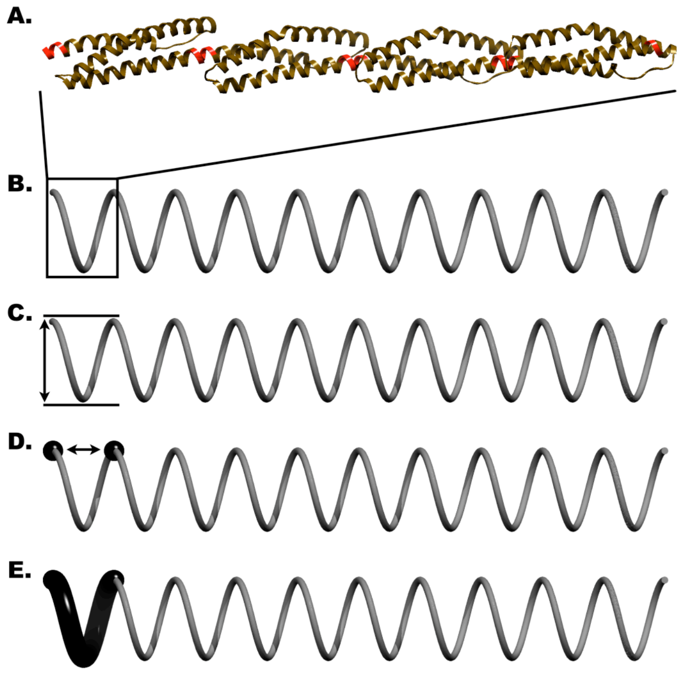

Supplemental Figure 3 *Geometric parameters used to describe spectrin***.** **A**. Ribbon representation of four spectrin repeats in the "Extended Model." **B**. Four of these repeats are arranged around each helical turn. **C**. The diameter of the helix is simply defined as the helical width. **D**. The pitch is the shortest distance between two equally phased points, while (**E**) the contour length is the end-to-end distance around the helix between any two equivalently phased points.
