# Supplementary material for: The Physiological Molecular Shape of Spectrin: A Compact Supercoil Resembling a Chinese Finger Trap
Source: PLoS Comput Biol. 2015 Jun 11;11(6):e1004302. doi: 10.1371/journal.pcbi.1004302 (PMC4466138; doi:10.1371/journal.pcbi.1004302)
Supplement: S4 Fig — (PDF) [file pcbi.1004302.s004.pdf]

FQSAD[E]TGQDLVNANHEASDEVR - DLVASEGLFHSH[K]GLER; Charge: +5; m/z: 882.4223.

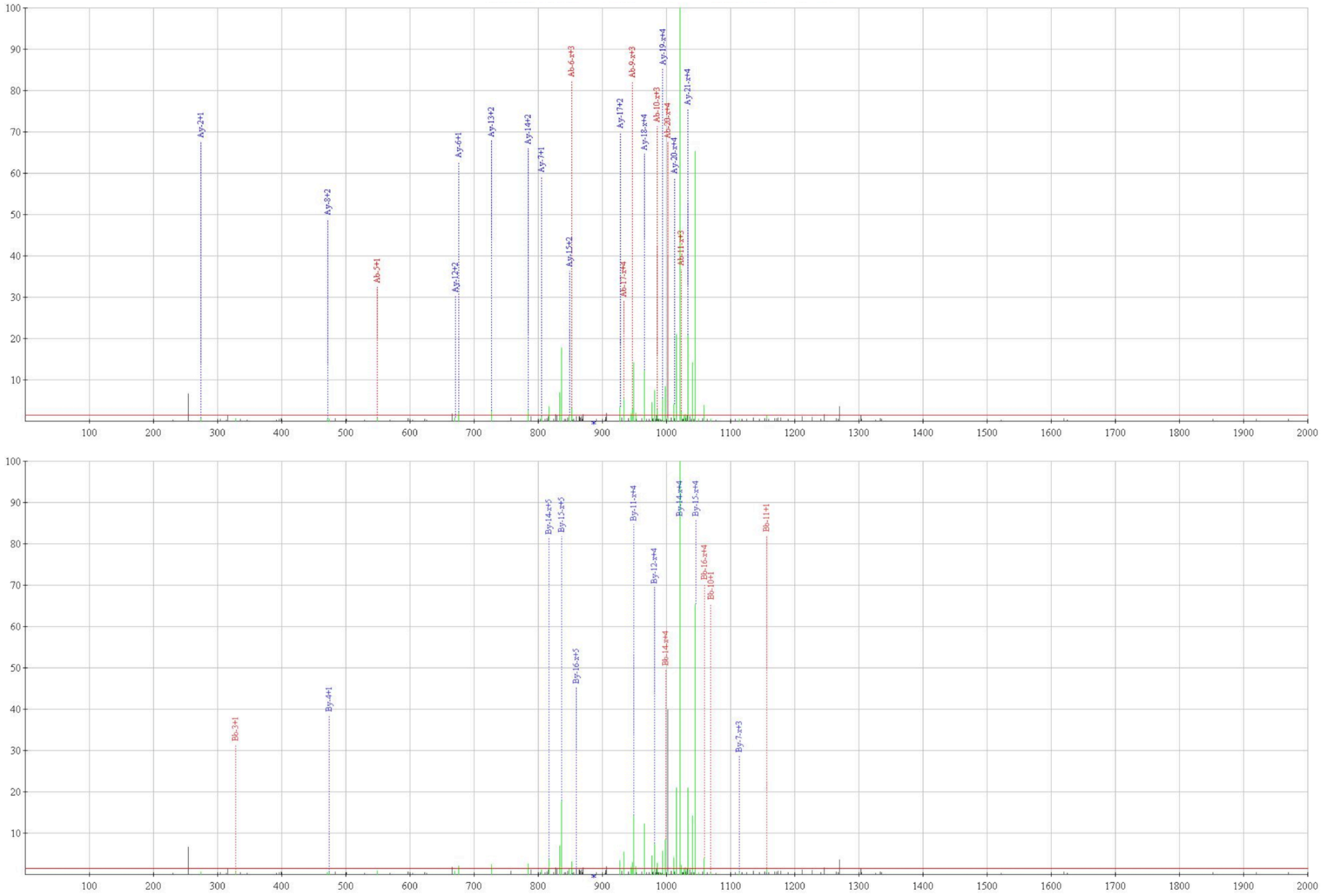

<u>Supplemental Figure 4</u> *Annotated MS/MS spectra of the K307-E431 cross-link generated by ZXMiner.* Peptide sequences along with location of identified b-ions and y-ions are at the top of each spectrum where red marks indicate ions that also contain the crosslinked site and intact second peptide and blue marks are regular b-ions and y-ions. Peaks colored in green are those that matched to theoretical ions. B-ions and y-ions are annotated in the spectrum with red and blue labels, respectively. Only major ions without neutral losses are shown. Red horizontal bars indicate the 5,000 ion counts intensity level. The top panel shows the contribution from the first peptide, and the bottom panel shows the contribution from the second peptide. Ion naming convention is as follow:

1. [Peptide][Ion Type]-[Ion Index]+[Charge] for regular ions. For example, Ab-10+2 represents a b-10 ion from peptide A (the first peptide) with charge state of +2.

2. [Peptide][Ion Type]-[Ion Index]-x+[Charge] for crosslinked ions. For example, Bb-10-x+4 represents a b-10 ion from peptide B (the second peptide) with charge state of +4 that also contains the crosslinked site and intact peptide A (the first peptide).
