# Supplementary material for: The Physiological Molecular Shape of Spectrin: A Compact Supercoil Resembling a Chinese Finger Trap
Source: PLoS Comput Biol. 2015 Jun 11;11(6):e1004302. doi: 10.1371/journal.pcbi.1004302 (PMC4466138; doi:10.1371/journal.pcbi.1004302)
Supplement: S5 Fig — (PDF) [file pcbi.1004302.s005.pdf]

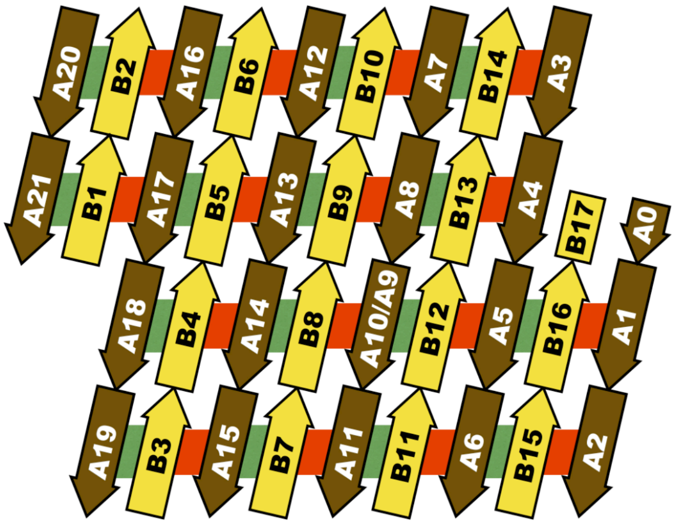

<u>Supplemental Figure 5</u> *Cartoon representation of a spectrin heterodimer in a compact supercoiled conformation*. This figure is identical to the left half of Figure 3C. The right side is omitted to emphasize that B17 and A0 are antiparallel to one another.
