# Supplementary material for: The Physiological Molecular Shape of Spectrin: A Compact Supercoil Resembling a Chinese Finger Trap
Source: PLoS Comput Biol. 2015 Jun 11;11(6):e1004302. doi: 10.1371/journal.pcbi.1004302 (PMC4466138; doi:10.1371/journal.pcbi.1004302)
Supplement: S6 Fig — (PDF) [file pcbi.1004302.s006.pdf]

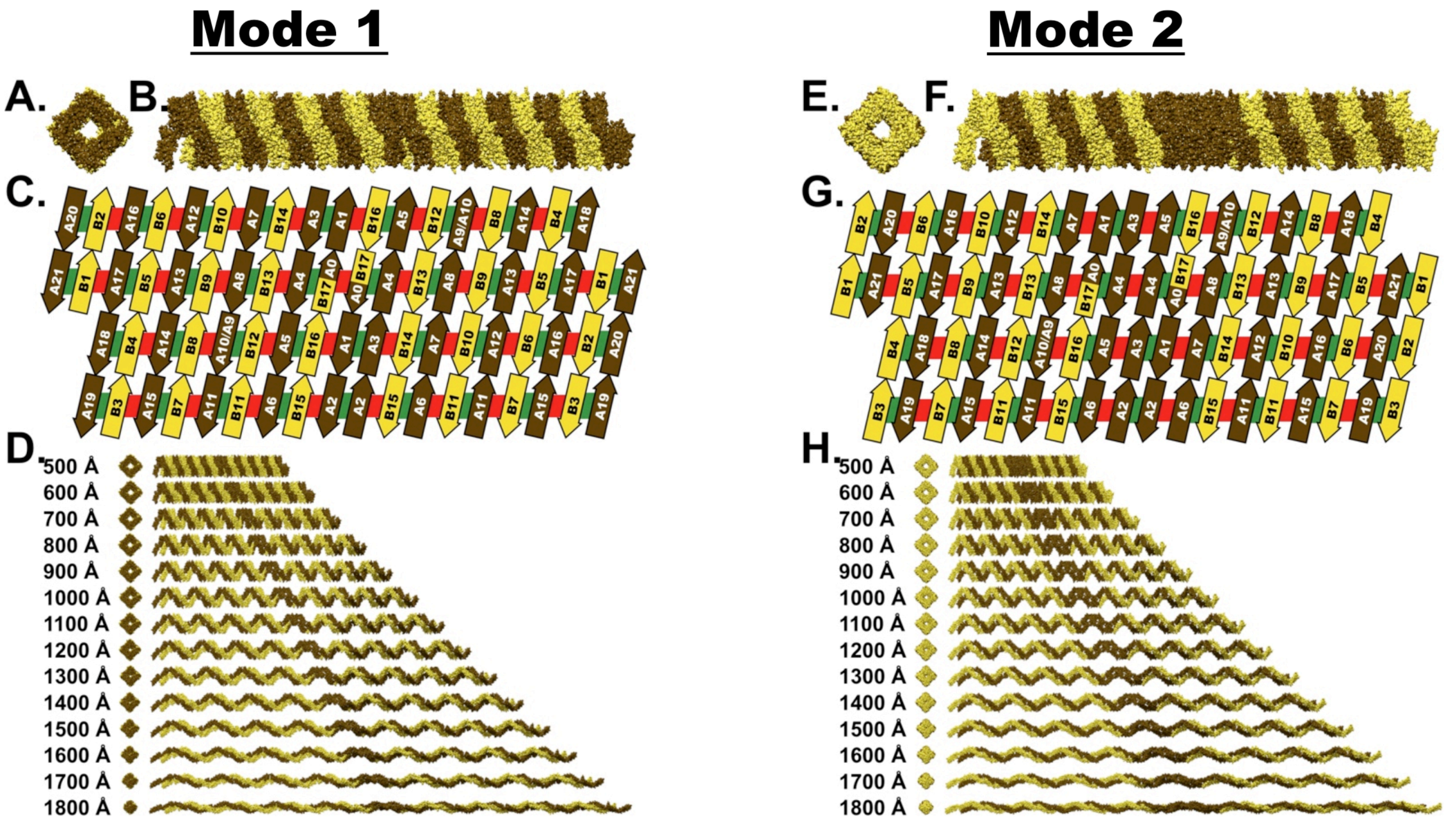

Supplemental Figure 6 *Comparison of the two potential antiparallel arrangements of the spectrin heterotetramers consistent with the Chinese Finger Trap model.* Both arrangements are two-start right-handed helices, the difference is whether alpha spectrin is lateral (Mode 1) or medial (Mode 2) to the beta spectrin. Another way of thinking of about these two modes is they can be interconverted by laterally translating one strand to the other side of its partner strand. The different symmetries of these two modes derives from the fact that the strands are antiparallel with respect to one another.
