# Supplementary material for: The Physiological Molecular Shape of Spectrin: A Compact Supercoil Resembling a Chinese Finger Trap
Source: PLoS Comput Biol. 2015 Jun 11;11(6):e1004302. doi: 10.1371/journal.pcbi.1004302 (PMC4466138; doi:10.1371/journal.pcbi.1004302)
Supplement: S7 Fig — (PDF) [file pcbi.1004302.s007.pdf]

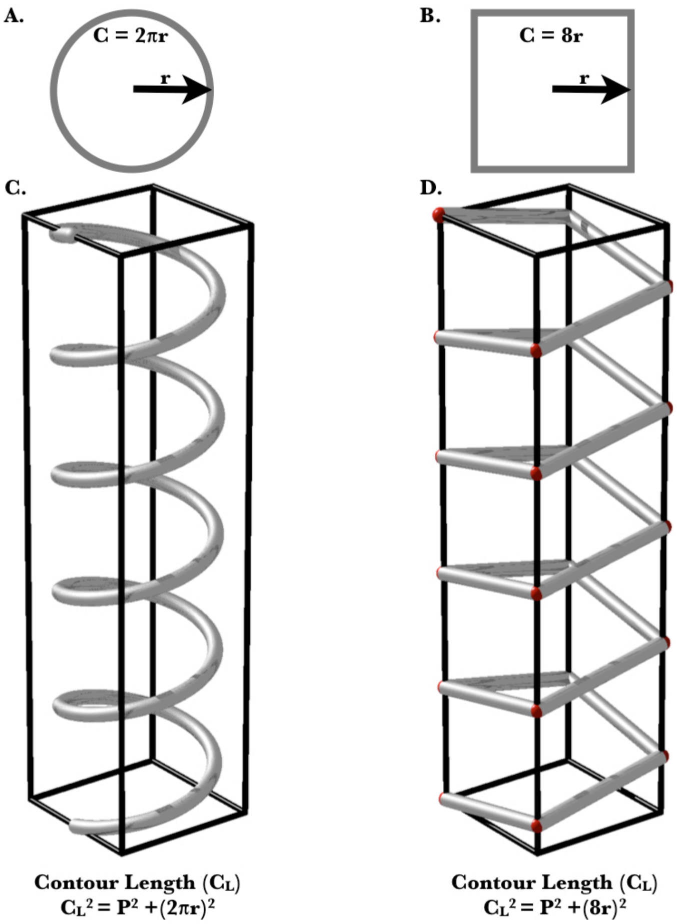

Supplemental Figure 7 *Comparison of the arrangement of spectrin repeats in the previous model (McGough & Josephs, 1990) to that of the Chinese Finger Trap model*. **A**, **C**. In the previous model each spectrin strand is organized as a continuous helical structure (depicted here as a chrome tube). **B**, **D**. In the Chinese Finger Trap model, spectrin repeats are modeled as rigid bars that are kinked at the interspersed linker region (red spheres) to assume a right-handed helical conformation.
