# Supplementary material for: The Physiological Molecular Shape of Spectrin: A Compact Supercoil Resembling a Chinese Finger Trap
Source: PLoS Comput Biol. 2015 Jun 11;11(6):e1004302. doi: 10.1371/journal.pcbi.1004302 (PMC4466138; doi:10.1371/journal.pcbi.1004302)
Supplement: S8 Fig — (PDF) [file pcbi.1004302.s008.pdf]

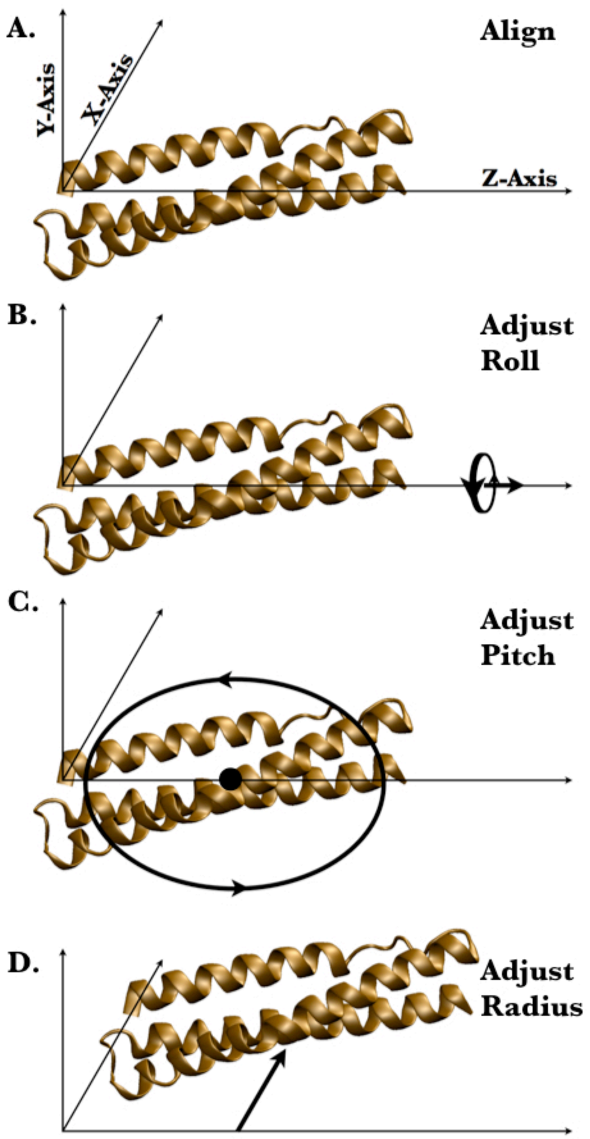

<u>Supplemental Figure 8</u> *Visual representation of the steps used to position the individual spectrin repeats in the Chinese Finger Trap model.* **A.** Each spectrin repeat is aligned along the Z-Axis. **B.** Next a "roll" is applied to each spectrin repeat. **C.** Next, a pitch is added to each spectrin repeat, which varies according to the final length of spectrin tetramer. **D.** Next, the spectrin repeat is translated perpendicular to the Z-Axis to add a radius. Like the pitch, this parameter varies according to the final length of the spectrin tetramer. After these adjustments have been implemented, the spectrin repeats are consecutively rotated in 90^0 increments about the Z-Axis and translated to the desired helical arrangement of the Chinese Finger Trap geometry.
