# Supplementary material for: The Physiological Molecular Shape of Spectrin: A Compact Supercoil Resembling a Chinese Finger Trap
Source: PLoS Comput Biol. 2015 Jun 11;11(6):e1004302. doi: 10.1371/journal.pcbi.1004302 (PMC4466138; doi:10.1371/journal.pcbi.1004302)
Supplement: S1 Table — (PDF) [file pcbi.1004302.s009.pdf]

Table 1 Methodology: Computing a theoretical length of spectrin [illegible] cellular abundance of an erythrocytic cytoskeletal protein.

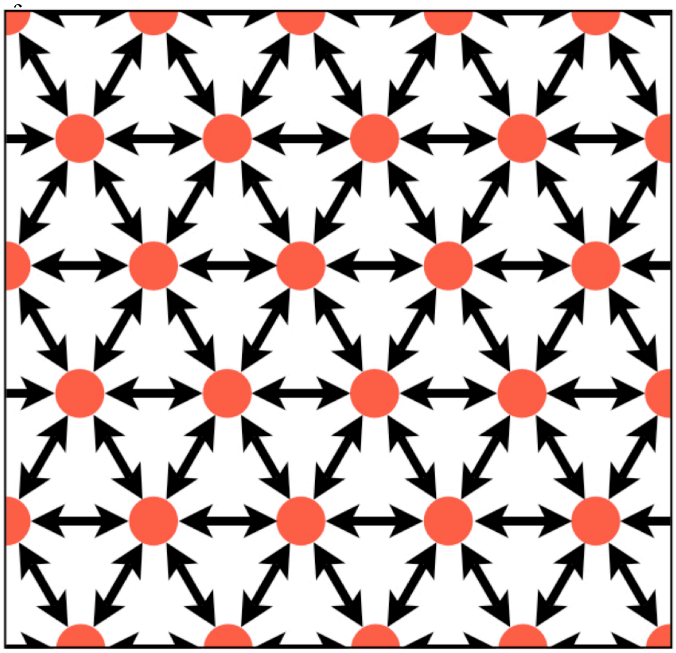

The erythrocytic cytoskeleton is composed of an approximately hexagonal network of spectrin crosslinked junctional complexes (1). This idealized structure is schematically represented to the right, where red circles are the junctional complexes that are cross-linked by spectrin tetramers (black arrows).

The fundamental unit of this hexagonal network is a triangle that contains 0.5 Junctional Complexes and 1.5 Spectrin Tetramers.

The erythrocyte is unique in that it has an extremely uniform size and shape with a surface area of 135×10^6 nm^2.

The structure, localization, and stoichiometry of the proteins that comprise the erythrocytic cytoskeleton have been reviewed several times in the literature as well as in textbooks. We used Bennett & Baines, 2001 (2) as our reference for the stoichiometry of the protein components. This reference details the position and stoichiometry of all components listed in Table 1 (Actin, Adducin, Ankyrin, Spectrin, Tropomyosin, and Tropomodulin) with the exception of dematin.

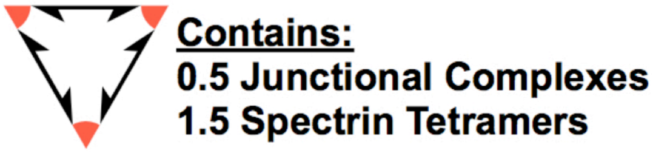

Dematin could not be included in this model because two critical studies published in the last 2 years were required to elucidate its function within the junctional complex. In 2012, Koshino *et al.* demonstrated that dematin acts as a cross-linker between spectrin and actin, stabilizing this interaction (3). When these data are viewed in light of our recent analytical ultracentrifugation data, which demonstrated that dematin is monomeric (4) as opposed to trimeric, the localization of dematin within the junctional complex becomes clearer. Dematin likely forms a ternary complex with spectrin and actin. If this interaction is fully saturated, it will result in 6 dematin molecules per junctional complex.

To calculate the length of spectrin from the cellular abundance of a cytoskeletal protein:

1) Divide the cellular abundance by the number of proteins expected to be in each triangle (see diagram). For components of the junctional complex, this will be 0.5 x the number of molecules per junctional complex. For ankyrin and spectrin this will be 1.5. This step will result in the theoretical number of triangles expected across the erythrocyte membrane.

2) Divide the surface area of erythrocyte (135×10^6 nm^2) by the calculated number of triangles. This will result in the theoretical area of each triangle.

3) Use the equation below to calculate the length of each side (length of spectrin) of an equilateral triangle from the theoretical area of the triangle calculated above.

$$\boldsymbol{Area} = \left(\boldsymbol{length}\right)^2 \frac{\sqrt{3}}{4}$$

References
1. Byers, T.J., Branton, D. (1985) Visualization of the Protein Associations in the Erythrocytic Membrane Cytoskeleton. *Proc. Natl. Acad. Sci. USA* **82**, 6153-7.
2. Bennett, V., Baines, A.J. (2001) Spectrin and Ankyrin-Based Pathways: Metazoan Interventions for Integrating Cells into Tissues. *Physiol Rev* **81**, 1353-92.
3. Koshino, I., Mohandas, N., Takakuwa, Y. (2012) Identification of a Novel Role for Dematin in Regulating Red Cell Membrane Function by Modulating Spectrin-Actin Interaction. *J. Biol. Chem.* **287**, 35244-50.
4. Chen, L., Brown, J.W., Mok, Y.F., Hatters, D.M., McKnight, C.J. (2013) The Allosteric Mechanism Induced by Protein Kinase A (PKA) Phosphorylation of Dematin (Band 4.9) *J. Biol. Chem.* **288**, 8313-20.
